# Extracellular Vesicles Secreted by Corneal Epithelial Cells Promote Myofibroblast Differentiation

**DOI:** 10.3390/cells9051080

**Published:** 2020-04-26

**Authors:** Tina B. McKay, Audrey E. K. Hutcheon, James D. Zieske, Joseph B. Ciolino

**Affiliations:** Schepens Eye Research Institute of Massachusetts Eye and Ear, Department of Ophthalmology, Harvard Medical School, Boston, MA 02114, USA

**Keywords:** extracellular vesicles, cornea, myofibroblast, proteomics, exosomes, fibroblast, wound healing, cell–cell communication

## Abstract

The corneal epithelium mediates the initial response to injury of the ocular surface and secretes a number of profibrotic factors that promote corneal scar development within the stroma. Previous studies have shown that corneal epithelial cells also secrete small extracellular vesicles (EVs) in response to corneal wounding. In this paper, we hypothesized that EVs released from corneal epithelial cells in vitro contain protein cargo that promotes myofibroblast differentiation, the key cell responsible for scar development. We focused on the interplay between corneal epithelial-derived EVs and the stroma to determine if the corneal fibroblast phenotype, contraction, proliferation, or migration were promoted following vesicle uptake by corneal fibroblasts. Our results showed an increase in myofibroblast differentiation based on α-smooth muscle actin expression and elevated contractility following EV treatment compared to controls. Furthermore, we characterized the contents of epithelial cell-derived EVs using proteomic analysis and identified the presence of provisional matrix proteins, fibronectin and thrombospondin-1, as the dominant encapsulated protein cargo secreted by corneal epithelial cells in vitro. Proteins associated with the regulation of protein translation were also abundant in EVs. This paper reveals a novel role and function of EVs secreted by the corneal epithelium that may contribute to corneal scarring.

## 1. Introduction

Corneal scarring affects over 4.9 million people worldwide as a leading cause of blindness [1] and may develop in response to injury, infection, or genetic corneal dystrophies [2]. Corneal scarring decreases vision by inducing tissue opacity that blocks light from entering the eye, and frequently distorts the shape of the cornea, which results in irregular astigmatism and higher order aberrations. While these irregularities in the cornea can often be corrected by hard contact lenses, opacities within the tissue necessitate surgery, such as corneal transplantation, in order to restore the eye’s visual potential. However, corneal transplants only have a 20-year life expectancy, on average [3], and are associated with comorbidities, such as cataracts [4] and glaucoma [5]. Moreover, tissue access for corneal transplantation is limited worldwide [6]; therefore, the development of novel treatments to inhibit corneal scar formation and potentially reverse this damage remains an unmet global need.

Resident corneal cell populations (e.g., epithelium, keratocyte, neuronal, and immune cells) mediate the collective corneal tissue response to injury via autocrine and exocrine signaling that influences clinical outcome, such as corneal scar development, persistent pain, and ultimately tissue regeneration [7]. As the anterior cell surface of the eye, the corneal epithelium expresses various soluble mediators following wounding, including proinflammatory cytokines, growth factors, and matrix metalloproteinases, that diffuse to the stroma and influence keratocyte activation. The initial response to injury by the corneal epithelium has been associated with promotion of the development of myofibroblasts within the stroma, the source of scar development [8].

Extracellular vesicles (EVs) are known to play an important role in mediating cell–cell communication via the stabilization of labile factors secreted into the extracellular environment (i.e., peptides, proteins, lipids, and microRNA) [9,10]. The role of EVs in cell–cell communication has been heavily studied in cancer biology [11,12] and has recently become a growing area of interest in corneal tissue biology in relation to physiological and pathological responses to wound healing [13,14,15]. Early work from our laboratory provided some of the first evidence that EVs are released into the corneal stroma following a keratectomy (surgical excision of a portion of the cornea) [16]. Further investigations have examined the effects of EVs in the cornea in terms of neovascularization [17] and found that corneal wounding promotes EV release by the corneal epithelium [17]. Studies from others have suggested that limbal stromal cell-derived EVs may influence the proliferation and migration of limbal epithelial stem cells in vitro with varying responses depending on the cell source (e.g., healthy versus diabetic corneal limbal rims) [13]. Additional studies have shown improved corneal epithelial wound healing [18] and scarless stromal recovery post-injury in vivo following stimulation with mesenchymal stem cell-derived EVs [19]. These studies have revealed a novel application of EVs as therapeutic carriers to promote corneal tissue regeneration. However, the functional role of EVs secreted from the corneal epithelium during wound healing remains unknown. 

Clinical studies suggest that the corneal basement membrane is important in preventing or limiting corneal scar development following epithelial debridement (superficial removal of the epithelial layer) or keratectomy (removal of the epithelium and anterior stroma), respectively [20,21]. Interestingly, the severity of the wound directly influences the ability of EVs secreted by the epithelium to diffuse to the stroma. If the basement membrane is left intact during an epithelial debridement, EV migration into the stroma appears to be restricted [17]. In contrast, a keratectomy, which removes the basement membrane and anterior stroma, results in free dispersion of secreted epithelial-derived EVs to the stromal layers [17]. The lack of EV diffusion in the presence of the epithelial basement membrane suggests that EVs may bind directly to this layer, thereby limiting migration. These studies led us to the hypothesis that EVs secreted by the corneal epithelium may play an important role in promoting stromal scar development [22]. 

The objective of this paper was to delineate the mechanistic details regarding the potential role of EVs in promoting myofibroblast differentiation by corneal fibroblasts. Our current study examined EVs secreted by human corneal epithelial cells (hCE-TJs) in vitro and their functional effects on fibroblast contraction, proliferation, migration, and the phenotype. We also characterized the protein content of hCE-TJ-derived EVs using tandem mass tag (TMT)-plex labelling and proteomic analysis. This study identified a novel role for corneal epithelial cell-derived EVs as carriers for provisional matrix proteins and factors that promote myofibroblast differentiation. 

## 2. Materials and Methods

### 2.1. Cell Culture

#### 2.1.1. Human Corneal Epithelial Cells

The immortalized corneal epithelial cell line, hCE-TJ [23], and a green fluorescent protein (GFP)-labelled hCE-TJ cell line [24] (Figure S1), were cultured in complete epithelial medium [Keratinocyte-SFM (Gibco, Grand Island, NY, USA), 0.05 mg/mL bovine pituitary extract (Gibco), 5 ng/mL epithelial growth factor (Gibco), and 1× antibiotic-antimycotic (Gibco)] at 37 °C/5% CO_2_. hCE-TJ conditioned medium was collected from 90% confluent cultures every other day and stored at 4 °C until EV isolation (<2 days).

#### 2.1.2. Human Corneal Fibroblasts

Primary hCFs were isolated from human corneas provided by the National Disease Research Interchange (NDRI, Philadelphia, PA, USA), as previously described [25,26]. Briefly, the epithelial and endothelial layers were debrided using a dulled blade followed by cutting of the stromal tissue into small pieces (~2 mm × 2 mm) and placing 3–5 pieces in a T25 flask. Following adhesion of the tissue to the bottom of the flask, complete corneal fibroblast medium (10% fetal bovine serum (FBS: Atlanta Biologicals, Flowery Branch, GA, USA), Eagle’s Minimum Essential Medium (EMEM: ATCC, Manassas, VA, USA), and 1× antibiotic-antimycotic) was added, and the cultures were incubated for 2–4 weeks to allow for cell migration from the explant. 

#### 2.1.3. Two-Dimensional (2D) Conventional Cultures

Primary hCFs (passage 2–4) or hCE-TJs were seeded onto coated glass slides (Lab-Tek II Chamber Slide, Lab-Tek, Rochester, NY, USA) at a density of 10^6^ cells/well. PKH26-staining of hCE-TJs was performed according to the manufacturer’s directions (Figure S2, PKH26 Red Fluorescent Cell Linker Kit for General Cell Membrane: Sigma Aldrich, St. Louis, MO, USA). Debridement was performed on 100% confluent cultures of hCE-TJ seeded in 150-mm petri dishes by gently scraping the cell layer using a sterile P20 pipette tip (8 scratches per dish). The medium was collected at *t* = 24 h post-scraping and subjugated to EV isolation.

#### 2.1.4. Three-Dimensional (3-D) Stromal Cultures

Primary hCFs (passage 2–4) were seeded onto polycarbonate transwell plate membranes (24 mm diameter with 0.4 μm pore, Corning, NY, USA) at a density of 10^6^ cells/well in complete corneal fibroblast medium. The medium was supplemented with 0.5 mM 2-O-α-D-glucopyranosyl-L-ascorbic acid (Wako Chemicals, Richmond, VA, USA) at 24 h following seeding and maintained for 4 weeks, with medium changes every other day.

### 2.2. EV Isolation

EVs were isolated using standard ultracentrifugation step gradients based on published protocols [27,28]. Briefly, hCE-TJ-conditioned medium or complete corneal fibroblast medium for hCE-TJ-EV or FBS-EV isolation, respectively, was collected on ice and subjected to successive centrifugation steps using a Beckman Type 50.2 Ti Rotor (Beckman Coulter, Brea, CA, USA) in an ultracentrifuge (Beckman Coulter, Optima LE-80K Ultracentrifuge) at increasing speeds (300× *g*, 10 min; 2000× *g*, 10 min; 11,004× *g*, 30 min; 146,000× *g*, 70 min) at 4 °C. The resulting pellet was re-suspended in the commercial total exosome isolation reagent (Invitrogen, Carlsbad, CA, USA) and phosphate-buffered saline (PBS) in a 1:2 ratio and incubated overnight at 4 °C with rocking, followed by centrifugation at 10,000× *g* for 60 min at 4 °C (Eppendorf Centrifuge 5417R, rotor F45-30-11, Hamburg, Germany). The isolated EV pellet was stored at −20 °C until further use. 

### 2.3. EV-Labelling

Isolated EVs were fluorescently labelled using the red PKH26 lipophilic dye (PKH26 Red Fluorescent Cell Linker Kit for General Cell Membrane). The EV pellet was resuspended in Diluent C and mixed with PKH26-dye in Diluent C buffer at a ratio of 1:1 for 2 min at room temperature. Bovine serum albumin (BSA, 1% *w*/*v* in Diluent C: Sigma Aldrich) was then added to the EV suspension at an equal ratio per volume and subjected to ultracentrifugation (146,000× *g*, 70 min, 4 °C: Beckman Coulter, Optima TLX Ultracentrifuge, rotor TLA-120.2). The PKH26-labelled EV pellet was washed and resuspended in Diluent C and ultracentrifuged (146,000× *g*, 70 min, 4 °C) followed by repeated washing/centrifugation for 3 × total. EVs were filter-sterilized (0.20 μm pore) prior to use in cell culture.

### 2.4. Transmission Electron Microscopy (TEM) Analysis

EVs were fixed and imaged by TEM, as previously described [27]. Briefly, the EV pellet was resuspended in 4% *w*/*v* paraformaldehyde (PFA) in PBS (Polysciences Inc., Warrington, PA, USA) for 30 min at room temperature for fixation. A 5-μL solution of the fixed EV pellet was added to an EM grid followed by a 20-min incubation to allow EVs to adhere to the grid surface. The grids were then washed in drops of PBS to remove residual PFA (repeat 5×) followed by resuspension in 1% *v*/*v* glutaraldehyde in PBS for 5 min. Residual glutaraldehyde was removed by gently resuspending the grid in water (repeat 7×). The grids then were transferred to a uranyl oxalate solution followed by a 10-min incubation with a methyl cellulose solution for contrast. The grid was allowed to dry before TEM imaging (JEM-1220 TEM: JEOL USA, Peabody, MA, USA).

### 2.5. Western Blot

For EV and cytosolic fractions, isolated EVs or cells were lysed for 10 min on ice in radioimmunoprecipitation assay (RIPA) buffer containing 1× protease inhibitors (Santa Cruz Biotechnology, Dallas, TX, USA). Samples then were centrifuged at 12,000× *g* for 60 min at 4 °C (Eppendorf Centrifuge 5417R, rotor F45-30-11) to pellet insoluble debris. The supernatant was isolated, aliquoted, and stored at −20 °C until further processing. A bicinchoninic acid assay (BCA) was performed following the manufacturer’s protocol (Micro BCA Protein Assay Reagent Kit: Pierce, Rockford, IL, USA). Western blot analysis was performed on isolated protein fractions (50 μg protein) using an 8–16% Novex Tris-glycine gel (Invitrogen) under non-reducing conditions at 125 V for 1.5 hours and transferred onto a 0.45-μm nitrocellulose membrane (GE Healthcare, Munich, Germany) at 25 V for 1–2 h at 4 °C. Following blocking in 5% *w*/*v* BSA for 1 h at room temperature, the membrane was incubated with the following primary antibodies overnight at 4 °C with rocking: Mouse monoclonal anti-β-actin (1:1000, Sigma Aldrich) and rabbit anti-α-smooth muscle actin (α-SMA, 1:1000, Epitomics/Abcam, Cambridge, MA, USA). The secondary antibodies (1:2000, donkey anti-mouse IRDye 800CW: LI-COR Biosciences, Lincoln, NE, USA; and donkey anti-rabbit IRDye 680RD: LI-COR Biosciences) were incubated with the membrane at room temperature for 1–2 h followed by imaging using a fluorescence scanner (Odyssey v. 3.0, LI-COR Biosciences).

### 2.6. Stimulated Emission Depletion (STED) Nanoscopy

EVs isolated from GFP-labelled hCE-TJs were suspended in PBS, applied to the surface of a glass microscopy slide, and allowed to dry for 30 min at room temperature in the dark. A drop of mounting medium (ProLong Diamond, Invitrogen) was then added to the EV smear, covered with a 1.5 glass coverslip, and allowed to incubate overnight at room temperature to permit curing. Samples were imaged on an SP8 confocal microscope with an STED 592 or 660 laser (Leica Microsystems, Bannockburn, IL, USA).

### 2.7. Immunofluorescence Microscopy

Samples were isolated and fixed in 4% *w*/*v* PFA in PBS for 10 min at room temperature, followed by permeabilization in 0.1% *v*/*v* Triton-X-100 (Sigma Aldrich) for 5 min, and blocking in 2% *w*/*v* BSA for 1 h. The following primary antibodies were incubated with each sample overnight at 4 °C with rocking, followed by an additional overnight incubation with the following corresponding secondary antibody, conjugated phalloidin, and/or nuclear stain: Mouse anti-α-SMA (1:25, Dako North America, Carpinteria, CA, USA), mouse anti-Ki67 (0.5 mg/L pre-diluted, Invitrogen), TRITC-conjugated donkey anti-mouse rhodamine (1:100, Jackson ImmunoResearch), fluorescein-phalloidin (1:40, ThermoFisher, Waltham, MA), rhodamine-phalloidin (1:40, ThermoFisher), and/or TOPRO-3-iodide (1:100, Life Technologies, Carlsbad, CA, USA). Samples were mounted with Vectashield Mounting Medium for Fluorescence (for samples with TOPRO-3) or Vectashield Mounting Medium for Fluorescence with DAPI (4′,6-diamidino-2-phenylindole) (Vector Laboratories, Burlingame, CA, USA) and imaged on a Leica SP5 confocal microscope (Leica Microsystems).

### 2.8. EV Uptake

PKH26-stained EVs were resuspended in complete corneal fibroblast medium and added to hCFs in 2-D culture for 12–16 h at 37 °C/5% CO_2_. Cultures were then isolated, washed with PBS 3× to remove unbound EVs, stained, and imaged by confocal microscopy. Quantification of EV uptake was performed on 10× images of each well using ImageJ (ImageJ 1.52a, National Institutes of Health, Bethesda, MD, USA) [29] and particle analysis with an adjusted threshold, circularity (0.00–1.00), and no size exclusion (0–∞).

### 2.9. Analysis of F-Actin Organization

The ImageJ plug-in, FibrilTool [30], was utilized to quantify the average angle and anisotropy of F-actin bundles based on the fluorescence signal detected using rhodamin-phalloidin staining. Briefly, a 40× fluorescent image detected in the red channel (λ_ex_ 550 nm, λ_em_ 573 nm) was opened in Fiji [31] and the region of interest was defined using the polygon tool, excluding areas with no signal. The average orientation of fibers (−90° to 90°) and anisotropy (1 to 0) detected within the sample were recorded using the ‘FibrilTool’ function [30]. 

### 2.10. Collagen Contraction Assay

Primary hCFs (passage 3) were trypsinized and resuspended in complete corneal fibroblast medium. A 7.2 × 10^4^ cells/mL suspension was added to 1 mg/mL rat-tail collagen type I (3 mg/mL stock, Gibco) neutralized with 6 μL of 1 M sodium hydroxide (Sigma Aldrich), and mixed by trituration. For EV-suspended gels, 25 μg/mL of EVs were added to the collagen mixture and mixed by trituration. Each cell/collagen mixture was then quickly added to each well of a 24-well plate and incubated for 20 min at room temperature followed by 20 min at 37 °C/5% CO_2_ to allow for gelation. The gel was disassociated from the sides of the well using a sterile pipette tip and incubated with complete corneal fibroblast medium overnight at 37 °C/5% CO_2_. Brightfield images of the collagen gels were taken on an LG Android (LG-D321: LG Electronics, Seoul, South Korea) from 0–334 h. For quantification, the relative diameter of the gel and well were measured for each image using ImageJ [29]. The percent change in the normalized ratio of well/gel area was calculated based on the change in gel area over time according to the following equation: [area at time (0 h) − area at time (X)]/[area at time (0 h)] × 100.

### 2.11. Proliferation Assay

Primary hCFs (passage 4) were grown to 30% confluence on glass slides (Lab-Tek II Chamber Slide, Lab-Tek) in complete corneal fibroblast medium. At 24 h post-seeding, cells were treated with PBS or hCE-TJ-derived EVs (30 μg total protein) for 24 h at 37 °C/5%CO_2_ followed by isolation and staining for Ki67 expression. ImageJ was used to count the cells in each sample [29], and the percent of Ki67-positive cells was determined based on the number of cells containing Ki67 staining divided by the total number of cells within the region of interest. 

### 2.12. Migration Assay

Primary hCFs (passage 4) were seeded into 24-well plates at a cell density of 2.5 × 10^4^ cells/well in complete corneal fibroblast medium. At 24 h post-seeding, a scratch wound was induced using a sterile P20 pipette tip. Brightfield images were taken at 0 and 24 h post-scratching using an inverted microscope with a 10 × objective lens (EVOS XL Core Imaging System: Life Technologies, Bothell, WA, USA).

### 2.13. Proteomics

Digestion, TMT-plex labelling, and mass spectrometry of isolated EVs for the quantification and validation of protein identities was performed by the Harvard Core Mass Spectrometry facility (Cambridge, MA, USA) based on standardized approaches in the field [32,33,34]. Only proteins identified in all three biological replicates were included for analysis, and proteins labelled as common contaminating proteins were excluded (e.g., keratin type II, trypsin, serum albumin precursor, fibrinogen, vitamin D-binding protein, apolipoprotein A, gelsolin, and transthyretin). The proteins were grouped and classified based on the following cellular localizations: 1) Cytoplasm (soluble); 2) cytoplasm (cytoskeleton/motor); 3) cytoplasm (endosomal trafficking); 4) extracellular (secreted); 5) nucleus; 6) ER/Golgi; 7) mitochondria; 8) plasma membrane; and 9) unknown. The cellular localization was defined based primarily on the UniProt Knowledgebase (https://www.uniprot.org/) [35] and the LOCATE subcellular localization database (http://locate.imb.uq.edu.au/) [36]. In addition, we utilized the Reactome Knowledgebase available at https://reactome.org to perform pathway analysis of EV-protein cargo [37,38].

### 2.14. Statistical Analysis

Statistical significance was assessed for multiple groups using a one-way or two-way analysis of variance (ANOVA) for column or grouped analysis, respectively. A comparison of two groups was analyzed using a Mann–Whitney t-test. All statistical analyses were performed in GraphPad Prism 7 for Windows (GraphPad Software, Version 7.03, La Jolla, CA, USA, www.graphpad.com). A *p*-value of ≤ 0.05 was considered statistically significant.

## 3. Results

### 3.1. Characterization of Epithelial-Derived EVs

**Figure 1:**
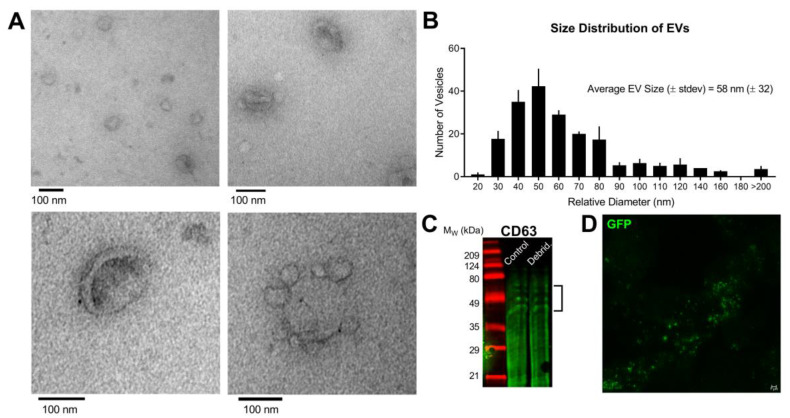
Relative morphology and size of EVs collected from hCE-TJ-conditioned medium. (**A**) TEM images of isolated EVs show the characteristic cup-shaped morphology of fixed EVs. Low-magnification (*top left panel*) and high-magnification (*top right and bottom panels*) show differential size ranges from 30–100 nm. (**B**) Size distribution of isolated EVs based on the relative diameter assessed by TEM. Data based on *n* = 3 independent EV preparations. Error bars represent the standard error of the mean (SEM). (**C**) CD63 protein expression in EVs isolated from conditioned medium of control and debrided hCE-TJ cell layers analyzed by Western blot under non-reducing conditions (predicted molecular weight of 30–60 kDa [42,45]). (**D**) Stimulated emission depletion (STED) microscopy of isolated GFP-hCE-TJ-derived EVs imaged under a 100× oil-immersion objective lens. Scale bar represents 1 μm.

We began by characterizing the biochemical and functional properties of EVs secreted by hCE-TJs in vitro in order to understand how epithelial-derived EVs influence wound healing. EVs were isolated from hCE-TJ-conditioned medium using established ultracentrifugation approaches [27] and identified EVs exhibiting the characteristic cup-like structure reminiscent of red blood cells (Figure 1A). The average size of hCE-TJ-derived EVs was 58 nm ± 32 nm, with a range of 20–340 nm. The most abundant size range of hCE-TJ-derived EVs was 50–59 nm, which is consistent with the relative size of exosomes or small microvesicles [10] (Figure 1B). We then compared EVs isolated from unwounded control versus debrided corneal epithelium, and verified the expression of a common EV marker, CD63 [39,40,41], in isolated EVs using Western blot (Figure 1C). Multiple bands were detected primarily from 45–60 kDa, consistent with the reported variations in the glycosylation of CD63 [42] (Figure 1C). We also applied a super-resolution microscopy approach, termed STED nanoscopy, as an additional method to visualize isolated EVs from GFP-expressing hCE-TJs. These GFP-loaded EVs appeared as single punctate particles, as well as in small aggregates with a strong fluorescent signal (Figure 1D). To determine the cytosolic localization of EVs in hCE-TJs, we utilized CD63 as a common marker for the exosomal surface [39,40,41]. In 2-D cultures of hCE-TJs, we identified the localization of CD63 primarily in the perinuclear region, which is the major site of exosomal loading and secretion via the endocytic pathway [43,44] (Figure S3).

### 3.2. EV Uptake by hCFs

**Figure 2:**
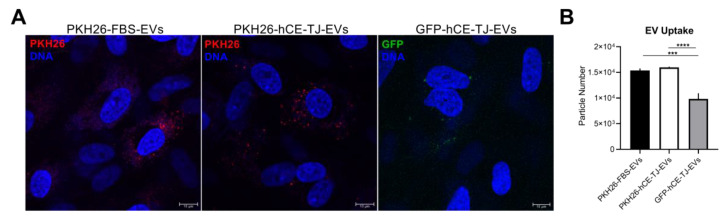
Uptake of EVs by human corneal fibroblasts (hCFs) in vitro occurs by 12–16 h post-application. (**A**) EVs isolated from FBS or hCE-TJ-conditioned medium were labelled with the lipophilic dye, PKH26, and applied topically to hCFs. EVs were also isolated from GFP-expressing hCE-TJ-conditioned medium and incubated with hCFs. Then, 25 μg/mL of EVs were applied to each sample. PKH26-labelled EVs = red; GFP-labelled EVs = green; Nuclei (DNA) = blue. Scale bars = 10 μm. (**B**) Quantification of EV uptake was performed using particle analysis in ImageJ based on the relative signal detected in the TRITC (λ_ex_ 550 nm, λ_em_ 573 nm) or FITC (λ_ex_ 495 nm, λ_em_ 517 nm) channel for hCFs containing PKH26- or GFP-labelled EVs, respectively. *n* = 6. Error bars represent SEM. *** *p* ≤ 0.001, **** *p* ≤ 0.0001 based on a one-way ANOVA.

EVs pre-stained with the lipophilic marker, PKH26, were added to hCFs in 2-D conventional cultures to determine whether EV uptake could be observed using confocal microscopy (Figure 2A). We identified the presence of endocytosed EVs by 12–16 h post-application. We compared the uptake of PKH26-labelled FBS-derived EVs and hCE-TJ-derived EVs, as well as GFP-hCE-TJ-derived EVs using particle analysis to determine if the cell source influenced the uptake by hCFs. The relative uptake of PKH26-labelled FBS and hCE-TJ EVs was similar; however, the number of endocytosed GFP-labelled EVs was significantly lower compared to FBS or hCE-TJ-derived EVs (36% and 39%, respectively; *p* ≤ 0.001, Figure 2B). This finding may be reflective of a lower percentage of total EVs isolated from GFP-hCE-TJs with detectable levels of GFP fluorescence.

### 3.3. Functional Effects of hCE-TJ-Derived EVs on hCFs 

**Figure 3:**
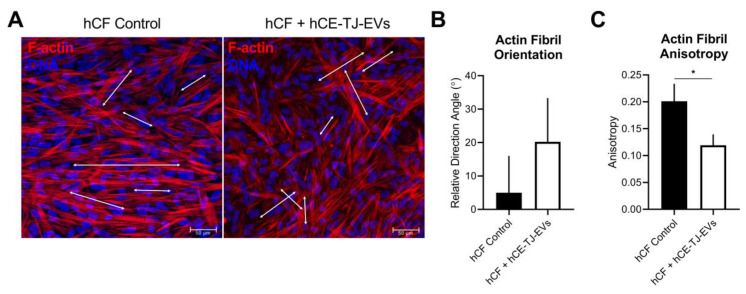
hCE-TJ-derived EVs promote decreased F-actin anisotropy in hCFs based on phalloidin staining. (**A**) Representative immunofluorescence images of hCF controls and hCFs treated with hCE-TJ-derived EVs. Then, 25 μg/mL of EVs were applied to each sample. Arrows depict the relative orientation and directionality of select actin fibrils within each group. Phalloidin (F-actin) = red; Nuclei (DNA) = blue. Scale bars = 50 μm. (**B**) The total actin fibril organization and (**C**) actin fibril fractional anisotropy quantified using the ImageJ plug-in, FibrilTool. *n* = 12 for contols and *n* = 13 for EV-treated groups. Error bars represent SEM. Statistical significance determined using a Mann–Whitney t-test. * *p* ≤ 0.05.

To determine if hCE-TJ-derived EVs promoted morphological changes in the cell structure, we analyzed the F-actin organization in the hCF control and EV-treated groups (Figure 3). We observed differences in the cell morphology in hCFs treated with hCE-TJ-derived EVs characterized by higher cell spreading compared to a more parallel cell alignment found in the hCF controls (Figure 3A). Quantification of the actin fibril orientation showed no significant difference in the directionality relative to the plane of view (ranging from −90° to 90° with respect to the x-axis) between control and EV-treated hCFs (4.98° ± 11.05° and 20.22° ± 13.11°, respectively, *p* = 0.320, Figure 3B). We then assessed the fractional anisotropy within groups, which is a measure of the disorder within the image with a range from 1 to 0, with 1 being completely ordered and 0 being a random distribution of actin fibrils. We found that hCFs treated with hCE-TJ-derived EVs exhibited significantly lower fractional anisotropy values compared to controls, suggesting that EV application stimulates changes in the cell cytoskeletal organization, thus affecting the total uniform directionality (0.201 ± 0.033 and 0.119 ± 0.020 for control and EV-treated groups, respectively, *p* = 0.036, Figure 3C).

**Figure 4:**
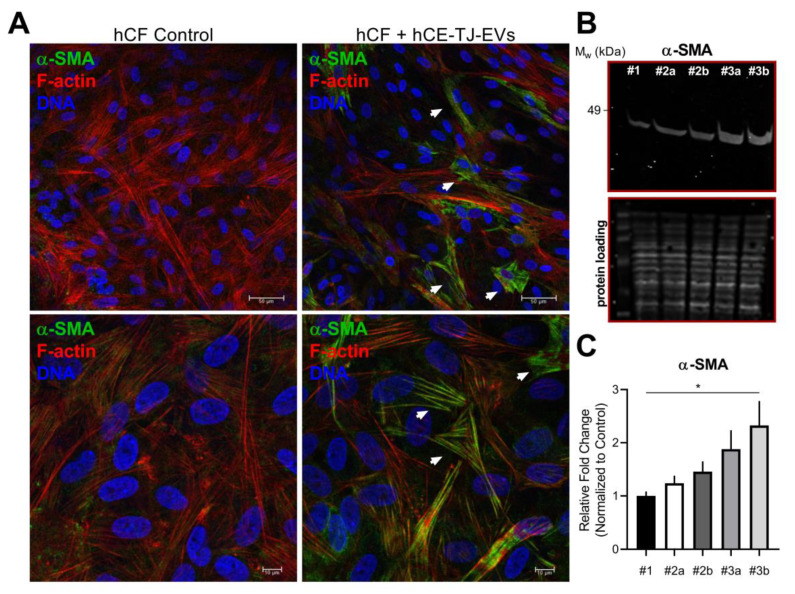
hCE-TJ-derived EVs increase stress fibers and protein expression of the myofibroblast marker, α-SMA, by hCFs. (**A**) Representative low-magnification (*top*) and high-magnification (*bottom*) immunofluorescence images of hCF controls and hCFs treated with hCE-TJ-derived EVs. Arrowheads denote the presence of stress fibers associated with elevated α-SMA expression. n=3. α-SMA = green; phalloidin (F-actin) = red; Nuclei (DNA) = blue. Scale bars = 50 μm (*top*) or 10 μm (*bottom*). (**B**) Western blot analysis of α-SMA in hCF control (#1), hCFs treated with hCE-TJ-derived EVs [#2a (non-debrided) and #2b (debrided)], and hCFs treated with hCE-TJ-conditioned medium containing both free- and EV-bound factors [#3a (non-debrided) and #3b (debrided)]. Predicted molecular weight (α-SMA) = 42 kDa. (**C**) Quantification of the α-SMA Western blot. *n* = 3 for control and *n* = 4 for each treated group. Each lane was loaded with 50 μg of total protein. Error bars represent SEM. Statistical analysis determined using a one-way ANOVA. * *p* ≤ 0.05.

The development of corneal scarring is largely dependent on the appearance and persistence of myofibroblasts, the contractile cell responsible for the secretion of a disorganized extracellular matrix (ECM) following wounding [8]. Based on the acute changes in F-actin distributions, we hypothesized that hCE-TJ-derived EVs may promote activation of hCFs to a more contractile phenotype. To determine if hCE-TJ-derived EVs promoted myofibroblast differentiation, we assessed the expression of α-SMA at 3 days following EV application and identified the presence of increased stress fibers in hCFs treated with hCE-TJ-derived EVs in select cells, with low expression apparent in controls (Figure 4A). Western blot analysis verified an increase in α-SMA expression in groups treated with hCE-TJ-conditioned medium, which contains both free- and EV-bound factors, exhibiting the highest increase in myofibroblast marker expression (1.88-fold (*p* = 0.19) and 2.33-fold (*p* = 0.032) for hCF + control and debrided hCE-TJ-conditioned medium, respectively, Figure 4B,C).

**Figure 5:**
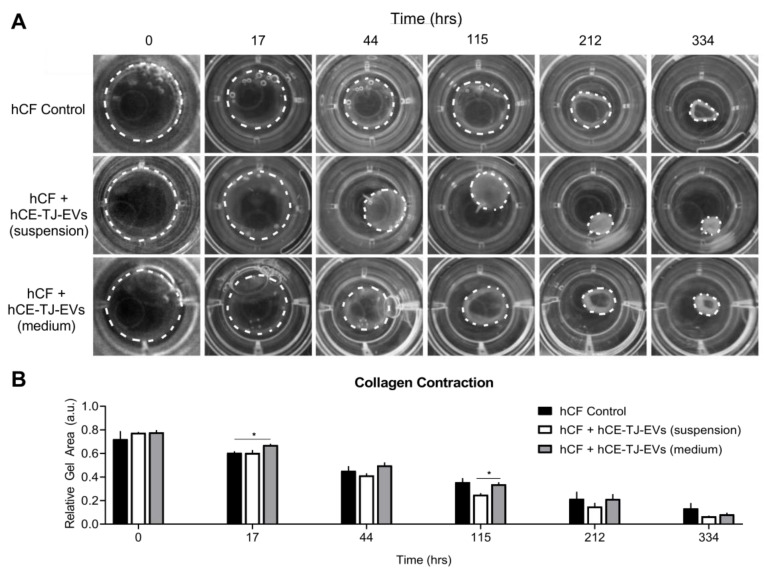
hCE-TJ-derived EVs increase corneal fibroblast contraction in floating collagen type I gels. (**A**) Representative brightfield images of floating hCF collagen gels over time. The dotted circle denotes the periphery of the collagen gel. hCFs suspended in collagen gels and maintained in complete fibroblast medium ± hCE-TJ-derived EVs for 0–334 h. Then, 25 μg/mL of EV protein was encapsulated within the collagen gel with hCFs (suspension) or included within the medium (medium). (**B**) Quantification of collagen contraction over time. *n* = 4. Error bars represent SEM. * *p* ≤ 0.05 based on a two-way ANOVA.

Given that hCE-TJ-derived EVs appeared to promote the conversion of fibroblasts to myofibroblasts, we sought to determine if cell contractility was affected by the presence of epithelial-derived EVs using a free-floating collagen gel. We compared three conditions: 1) Untreated hCFs; 2) hCF and hCE-TJ-derived EVs suspended within the collagen gel; and 3) hCFs with hCE-TJ-derived EVs present in the cell culture medium (Figure 5A). We hypothesized that EVs that have already been encapsulated throughout the gel would promote a faster rate of contraction compared to EVs dispersed within the medium and require diffusion through the hydrogel. At *t* = 17 h post-seeding, our results showed an initial delay in contraction in hCFs with hCE-TJ-EVs included within the medium compared to the control (10% decrease, *p* = 0.02, Figure 5B). Moreover, the inclusion of EVs in the medium promoted more contraction at the periphery of the gel compared to hCFs treated with EVs suspended within the collagen gel, which yielded a more uniform contraction (Figure 5A). Interestingly, hCFs with suspended EVs showed an increase in contractility that became apparent at *t* = 115 h and continued to 334 h (26%, *p* = 0.02, Figure 5B). These results are consistent in showing that hCE-TJ-derived EVs promote a contractile cell phenotype suggestive of a myofibroblast.

**Figure 6:**
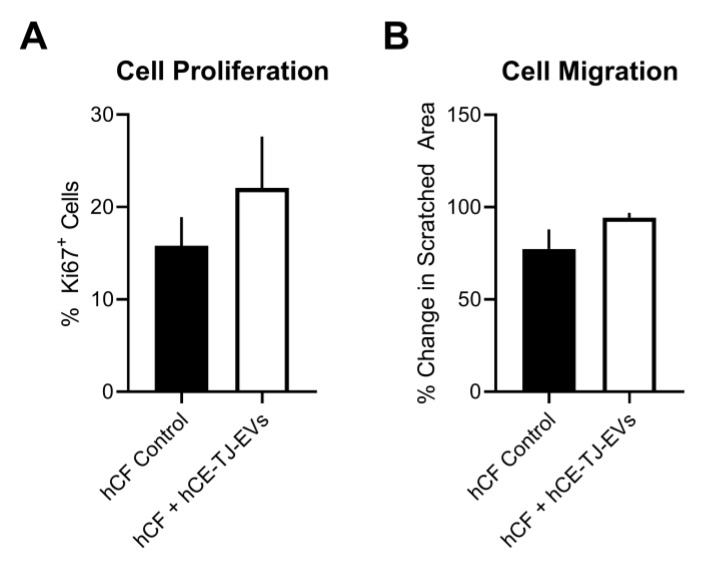
hCE-TJ-derived EVs do not significantly influence corneal fibroblast proliferation or migration. (**A**) hCF proliferation based on the marker of proliferation (Ki67^+^) staining at 24 h. *n* = 5–6. (**B**) Relative change in the wounded area in hCF ± hCE-TJ-EVs at 24 h. *n* = 3. Error bars represent SEM.

Corneal wound healing requires rapid cell proliferation and migration in order to repopulate the region of injury and reduce permanent tissue damage [7]. To determine if EVs that were taken up attenuated the proliferation of hCFs, we assessed the effects of hCE-TJ-derived EVs on Ki67 expression, which is a marker for cell proliferation [46], following 24 h post-EV stimulation. A modest, though not statistically significant, change in proliferation was observed by 24 h (% Ki67-positive cells ± SEM = 16% ± 3% and 22% ± 5% in control and EV-treated groups, respectively, *p* = 0.4, Figure 6A). Additional studies applying BrdU incorporation at a shorter timepoint (8 h) may be useful to identify if hCE-TJ-derived EVs promote entry into the S-phase of the cell cycle.

To determine if cell migration was mitigated in response to EVs, we performed a scratch assay and evaluated the cell layer recovery at 24 h post-wounding. Likewise, we found a slight increase in cell migration, though this effect appeared to be insignificant in hCFs treated with hCE-TJ-derived EVs compared to controls (% change in area ± SEM = 77% ± 11% and 94% ± 3% in control and EV-treated groups, respectively, *p* = 0.2, Figure 6B). Cell proliferation likely contributed to closure of the scratch wound; however, the Ki67 proliferation assay showed no significant difference between control and EV-treated groups, suggesting that EV treatment did not accelerate closure via cell division.

### 3.4. EV Protein Cargo

**Figure 7:**
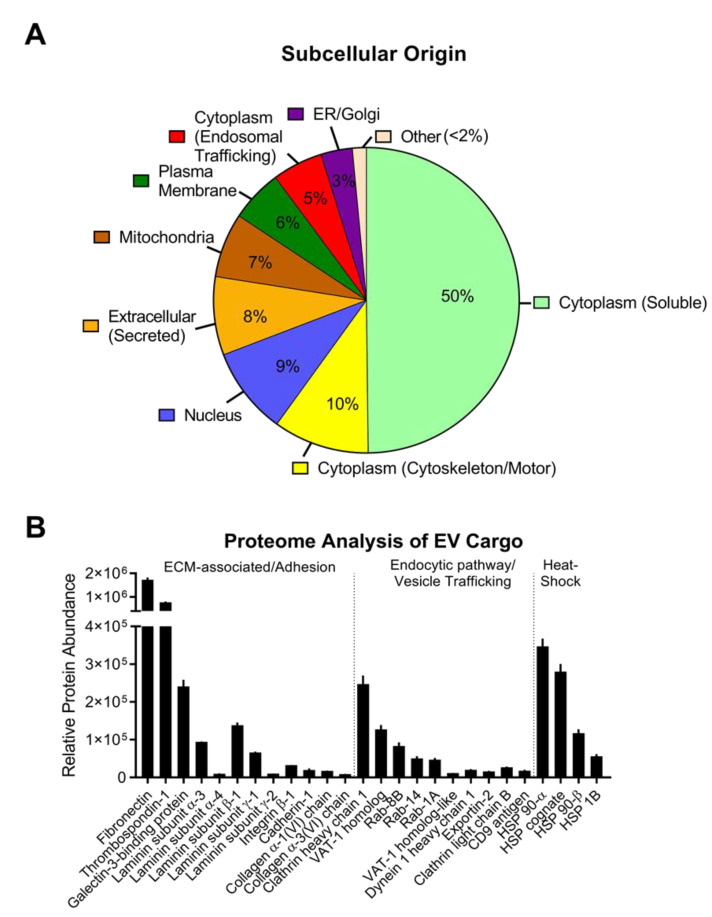
Protein cargo identified in unwounded hCE-TJ-derived EVs by proteomic analysis. (**A**) Proteins identified in isolated hCE-TJ EVs categorized by the dominant subcellular origin and classified based on nine major subcellular locations according to the UniProtKB database [53]. A full list of the proteomics data can be found in Table S1. (**B**) Distribution of select proteins identified in hCE-TJ-derived EV proteome. *n* = 3. Error bars represent SEM.

To determine the protein cargo found in corneal epithelial-derived EVs, we performed proteomic analysis on EVs derived from hCE-TJs cultured in vitro. We identified over 556 proteins isolated from each biological sample, suggesting that a broad distribution of factors were encapsulated in the secreted EVs (Figure 7A, Table S1). In addition, our results validated the presence of various proteins previously associated as exosomal markers, including cadherin and CD9 (Figure 7B). Of interest, ECM-associated proteins were the most abundant class identified in hCE-TJ-derived EVs, including fibronectin, thrombospondin, and laminin (Figure 7B). Cytosolic heat-shock proteins also contributed to the dominant class of proteins, along with endocytic/vesicle trafficking proteins (e.g., the Rab proteins and dynein). Importantly, very few growth factors were identified in secreted EVs, suggesting that the majority of proteins secreted by the corneal epithelium in response to injury, such as transforming growth factor-β, platelet-derived growth factor, interleukin-1, and tumor necrosis factor-α, are likely secreted as soluble proteins [47,48,49]. This mechanism is in agreement with studies showing that these growth factors bind directly to extracellular receptors found on the receiving cell membrane [50,51], rather than direct encapsulation via endocytosis. Thrombospondin-1, which is a known profibrotic factor that may activate latent transforming growth factor-β1 by binding to the latency-activating protein [52], was highly abundant in hCE-TJ-derived EVs. This data suggests that EV-packaged thrombospondin-1 secreted by hCE-TJs may promote stromal fibroblasts to differentiate to myofibroblasts. Furthermore, we compared control and debrided hCE-TJ and identified no significant difference in the EV protein cargo between hCE-TJ controls and scratched hCE-TJ, suggesting that in vitro debridement alone does not induce significant changes in the EV content (Figure S5).

We performed a post-processing examination of our data using a proteome analysis program known as Reactome [37,38] to determine a genome-wide overview of the pathways potentially regulated by hCE-TJ-derived EV protein cargo. We found that the following significant pathways were also regulated by hCE-TJ-derived EV proteins: The formation of the 43S preinitiation complex, which is involved in the initial steps of protein translation; PAK-2p34 regulation; AUF1 binding and mRNA stabilization; and translation initiation complex formation (Table 1). Interestingly, pathways related to translation and elongation, peptide chain elongation, and translation termination were also regulated by the proteins detected in hCE-TJ-derived EVs (Table 1).

A number of proteins (67 out of 294) associated with protein translation were detected in hCE-TJ-derived EVs. These proteins included amino acid-conjugated tRNA ligases, e.g., alanine-, asparagine-, aspartate-, glycine-, serine-, threonine-, and tryptophan-tRNA ligase, along with eukaryotic translation initiation factor and elongation factors (Table 2). The most abundant amino acid-linked tRNAs were alanine- and glycine-tRNA ligase (1.0 × 10^5^ ± 2.0 × 10^4^ and 1.0 × 10^5^ ± 9.4 × 10^3^, respectively) compared to the less abundant aspartate- and tryptophan-tRNA ligases (5.9 × 10^3^ ± 8.8 × 10^2^ and 6.4 × 10^3^ ± 8.3 × 10^2^, respectively, Table 2).

### 3.5. hCE-TJ-Derived EV Protein Localization

**Figure 8:**
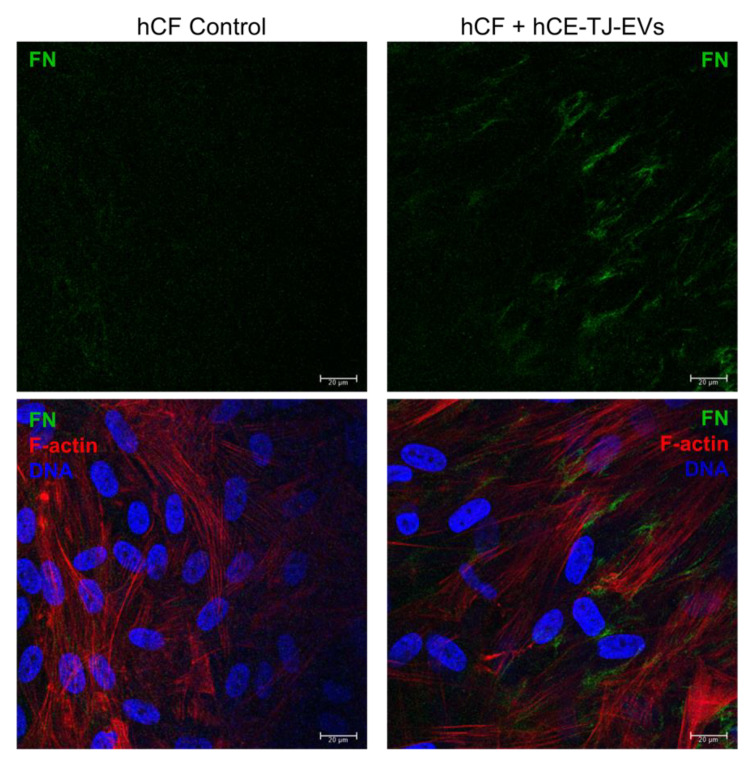
Increased fibronectin localization in hCF constructs with hCE-TJ-derived EV treatment at 3 days post-stimulation. Representative images based on *n* = 3. Fibronectin (FN) = green; phalloidin (F-actin) = red; Nuclei (DNA) = blue. Scale bars = 20 μm.

The significant abundance of provisional matrix proteins, such as fibronectin, led us to the question of whether hCE-TJ-EV application promoted the accumulation of fibronectin within the 3-D construct. Therefore, we treated hCFs in 3-D constructs with hCE-TJ-derived EVs and isolated the samples at 3 days to observe fibronectin localization (Figure 8). Fibronectin expression was 22% lower in controls, while hCE-TJ-derived EV-treated hCFs showed an accumulation of fibronectin along the external cell body with a fibrillar staining pattern. Previous literature has indicated that fibronectin is upregulated following corneal injury (e.g., a keratectomy) in vivo, with the accumulation of this provisional matrix protein along the epithelial–stromal interface [16,54]. Our results suggest that EVs containing fibronectin may accumulate on the surface of stromal hCFs or may also promote increased secretion of this protein.

## 4. Discussion

The results of our study indicate that EVs secreted by corneal epithelial cells are bioactive and may promote myofibroblast differentiation, which further supports the growing evidence for the importance of cell–cell communication between the corneal epithelium and stroma following injury. This study characterized the functional effects of hCE-TJ-derived EVs on corneal fibroblasts, showing the promotion of α-SMA expression and increased contractility with little change in proliferation or migration. We further identified the dominant protein cargo contained in hCE-TJ-derived EVs, showing the regulation of pathways associated with translation and cell cycle control. We identified no significant difference in the protein cargo of EVs isolated from unwounded versus debrided hCE-TJ cultures, suggesting that the process of partial removal of the cell layer in vitro does not appear to drastically alter EV packaging or secretion. However, debridement of a confluent epithelial layer in vitro involves the disruption of cell junctions and is a relatively modest wound compared to debridement of the corneal epithelium in vivo, which involves damage of the nerve fiber endings and stimulation of possible immune cell activation [55,56]. Further studies are required to determine the effects of wounding on EV protein cargo following corneal epithelial debridement and keratectomy in the complex in vivo microenvironment. 

Previous work identified that corneal epithelium-derived EVs are restricted by the basement membrane in vivo [17]. Our results suggest that EVs may be an important modality for regulating the stromal response during homeostasis or wounding by promoting the activation of resident corneal fibroblasts. Thus, limiting the diffusion of these EVs may reduce scar development in circumstances where the epithelial basement membrane is damaged [21].

Proteomic analyses of cancer models [57,58], leukocytes [59], and biological fluids [27] have identified a diverse distribution of proteins found in isolated exosomes. Interestingly, numerous reports have shown that EVs contain "essential" proteins reflective of the cytosolic environment, including actin, tubulin, adhesion-related molecules, heat shock proteins, metabolic enzymes, ribosomal, and Ras signaling proteins (reviewed in [60]). These results and the conservation between species have led to suggestions of a "life-preserving" function of EVs dependent on the cell type that may mediate the required activities for proper physiological function from the host to the recipient cell [61]. Our results showed an abundance of these proteins, in addition to exosomal trafficking proteins and provisional matrix proteins, suggesting that the protein cargo contained in EVs that are taken up may directly influence the cytosolic contents of the recipient stromal fibroblast. A question remains of whether some of the proteins identified, such as cytoskeletal or transcription factors, which are normally associated with cytosolic or nuclear localization, are encapsulated in vacuoles serendipitously as a result of adhesion to lipid molecules, or rather funneled into the endocytic pathway and multivesicular bodies for active secretion. 

Previous studies have identified that fibronectin-associated EVs may play an important role in influencing cell migratory behavior (i.e., directionality and speed) via interactions with cell integrins and adhesive proteins [62,63]. Further studies to determine whether the EV-associated fibronectin detected in our study influences epithelial cell migration following corneal injury are warranted. It is clear that a diverse range of functions of EVs in the cornea are likely involved in wound repair. Our study focused on an important aspect in corneal wound healing involving factors that mediate tissue closure (via the generation of myofibroblasts). However, the EV-bound factor secreted by corneal epithelial cells involved in promoting myofibroblast differentiation remains uncertain. Of note, we detected significant amounts of thrombospondin-1, which is considered a profibrotic factor as an activator of latent transforming growth factor-β1 [52], and may serve to promote myofibroblast differentiation by corneal fibroblasts. It is possible that the stromal response promoted by corneal epithelial-derived EVs may also be influenced by microRNA or the combination with protein content (free forms and encapsulated in EVs), rather than being solely protein derived. Identification of the effects of EVs secreted by the corneal epithelium may allow for new treatments that aim to reduce scar development of the cornea following epithelial injury.

## 5. Conclusions

Our results identified a potentially novel role for epithelial cell (hCE-TJ)-derived EVs in promoting the differentiation of corneal fibroblasts to myofibroblasts. The dominant proteins present in hCE-TJ-derived EVs consist of provisional matrix proteins, e.g., fibronectin and thrombospondin-1, as well as pathways associated with protein translation initiation, elongation, and termination. Further studies are required to determine whether epithelial-derived EV protein cargo is altered in response to injury in an in vivo model.

## Figures and Tables

**Table 1:** List of the top 25 pathways associated with proteins present in unwounded hCE-TJ-derived EVs. Results were obtained from https://reactome.org based on input of the accession number of proteins detected by proteomics.

Pathway Name	Entitites				Reactions	
	Found	Ratio	*p*-Value	FDR *	Found	Ratio
Formation of the ternary complex, and subsequently, the 43S complex	28/52	0.005	1.11 × 10^−16^	3.11 × 10^−15^	3/3	2.44 × 10^−4^
Regulation of activated PAK-2p34 by proteasome mediated degradation	26/50	0.004	1.11 × 10^−16^	3.11 × 10^−15^	2/2	1.62 × 10^−4^
AUF1 (hnRNP D0) binds and destabilizes mRNA	29/56	0.005	1.11 × 10^−16^	3.11 × 10^−15^	4/4	3.25 × 10^−4^
Translation initiation complex formation	30/59	0.005	1.11 × 10^−16^	3.11 × 10^−15^	2/2	1.62 × 10^−4^
Ribosomal scanning and start codon recognition	30/59	0.005	1.11 × 10^−16^	3.11 × 10^−15^	2/2	1.62 × 10^−4^
Eukaryotic Translation Elongation	42/95	0.008	1.11 × 10^−16^	3.11 × 10^−15^	9/9	7.31 × 10^−4^
Peptide chain elongation	39/90	0.008	1.11 × 10^−16^	3.11 × 10^−15^	5/5	4.06 × 10^−4^
Formation of a pool of free 40S subunits	43/102	0.009	1.11 × 10^−16^	3.11 × 10^−15^	2/2	1.62 × 10^−4^
GTP hydrolysis and joining of the 60S ribosomal subunit	47/113	0.01	1.11 × 10^−16^	3.11 × 10^−15^	3/3	2.44 × 10^−4^
Nonsense-Mediated Decay (NMD) independent of the Exon Junction Complex (EJC)	38/96	0.01	1.11 × 10^−16^	3.11 × 10^−15^	1/1	8.12 × 10^−5^
Viral mRNA Translation	37/101	0.009	1.11 × 10^−16^	3.11 × 10^−15^	2/2	1.62 × 10^−4^
Nonsense-Mediated Decay (NMD)	40/117	0.01	1.11 × 10^−16^	3.11 × 10^−15^	6/6	4.87 × 10^−4^
Nonsense-Mediated Decay (NMD) enhanced by the Exon Junction Complex (EJC)	40/117	0.01	1.11 × 10^−16^	3.11 × 10^−15^	5/5	4.06 × 10^−4^
SRP-dependent cotranslational protein targeting to membrane	37/113	0.01	1.11 × 10^−16^	3.11 × 10^−15^	5/5	4.06 × 10^−4^
Cap-dependent Translation Initiation	47/120	0.011	1.11 × 10^−16^	3.11 × 10^−15^	17/18	0.001
Eukaryotic Translation Initiation	47/120	0.011	1.11 × 10^−16^	3.11 × 10^−15^	19/21	0.002
Activation of the mRNA upon binding of the cap-binding complex and eIFs, and subsequent binding to 43S	30/60	0.005	1.11 × 10^−16^	3.11 × 10^−15^	5/6	4.87 × 10^−4^
Regulation of Apoptosis	26/53	0.005	1.11 × 10^−16^	3.11 × 10^−15^	4/5	4.06 × 10^−4^
Eukaryotic Translation Termination	38/94	0.008	1.11 × 10^−16^	3.11 × 10^−15^	4/5	4.06 × 10^−4^
Hh mutants that do not undergo autocatalytic processing are degraded by ERAD	26/56	0.005	1.11 × 10^−16^	3.11 × 10^−15^	4/6	4.87 × 10^−4^
L13a-mediated translational silencing of Ceruloplasmin expression	47/112	0.01	1.11 × 10^−16^	3.11 × 10^−15^	2/3	2.44 × 10^−4^
Host Interactions of HIV factors	41/144	0.013	1.11 × 10^−16^	3.11 × 10^−15^	34/54	0.004
Translation	67/294	0.026	1.11 × 10^−16^	3.11 × 10^−15^	57/99	0.008
Hh mutants abrogate ligand secretion	26/59	0.005	1.11 × 10^−16^	3.11 × 10^−15^	4/7	5.69 × 10^−4^
Ubiquitin-Mediated Degradation of Phosphorylated Cdc25A	25/52	0.005	1.11 × 10^−16^	3.11 × 10^−15^	2/4	3.25 × 10^−4^

* false discovery rate.

**Table 2:** Key proteins associated with protein translation identified in unwounded hCE-TJ-derived EVs.

Protein Name	UniProt Id	Relative Protein Abundance [Average (± Stdev)]
Alanine-tRNA ligase	P49588	1.0 × 10^5^ (± 2.0 × 10^4^)
Aminoacyl tRNA synthase complex	Q12904	2.2 × 10^4^ (± 3.4 × 10^3^)
Asparagine-tRNA ligase	O43776	1.6 × 10^4^ (± 4.1 × 10^3^)
Aspartate-tRNA ligase	P14868	5.9 × 10^3^ (± 8.8 × 10^2^)
Elongation factor 1-alpha 1	P68104	5.2 × 10^5^ (± 3.6 × 10^4^)
Elongation factor 1-gamma	P26641	2.9 × 10^5^ (± 2.3 × 10^4^)
Elongation factor 2	P13639	3.6 × 10^5^ (± 4.3 × 10^4^)
Eukaryotic translation initiation factor 3 subunit F	O00303	2.1 × 10^4^ (± 4.2 × 10^3^)
Glycine-tRNA ligase	P41250	1.0 × 10^5^ (± 9.4 × 10^3^)
Serine-tRNA ligase	P49591	1.5 × 10^4^ (± 8.7 × 10^2^)
Threonine-tRNA ligase	P26639	2.6 × 10^4^ (± 3.8 × 10^3^)
Tryptophan-tRNA ligase	P23381	6.4 × 10^3^ (± 8.3 × 10^2^)

## Supplementary Materials

The following are available online at https://www.mdpi.com/2073-4409/9/5/1080/s1, Figure S1: Fluorescence microscopy images of GFP-labelled hCE-TJs cultured in 2-D monocultures shows high GFP expression; Figure S2: PKH26-stained hCFs show broad cytosolic fluorescent staining; Figure S3: Expression of exosomal markers, CD63 and CD9, in hCE-TJs cultured in 2-D monocultures; Figure S4: Representative brightfield images of the collagen contraction assay. Table S1: Proteins identified in isolated epithelial-derived (hCE-TJ) EVs categorized by dominant subcellular origin; Figure S5. Effects of epithelial cell layer (hCE-TJ)-debridement on EV-protein cargo. Brightfield images of control and debrided hCE-TJ monolayers (*left*). Heat map showing the relative protein expression of hCE-TJ control EVs and debrided hCE-TJ-derived EVs (*right*). Figure S6: Full Western blot of CD63 staining. Figure S7: Full Western blot of α-SMA staining. Figure S8: Black and white images of the particle analysis of the PKH26- or GFP-fluorescent signal for the EV uptake study. Figure S9: Stimulated emission depletion (STED) microscopy images of isolated GFP-expressing hCE-TJ-derived EVs.
