# Supplementary material for: Extracellular Vesicles Secreted by Corneal Epithelial Cells Promote Myofibroblast Differentiation
Source: Cells. 2020 Apr 26;9(5):1080. doi: 10.3390/cells9051080 (PMC7290736; doi:10.3390/cells9051080)
Supplement: Supplementary file 1 [file cells-09-01080-s001.pdf]

cells MDPI

Supplemental Materials

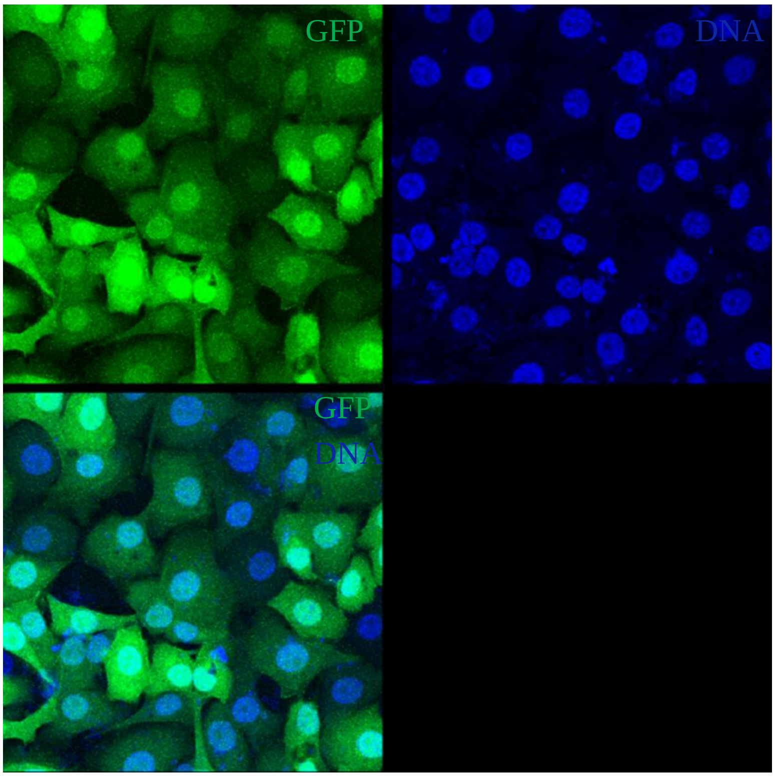

Figure S1. Fluorescence microscopy images of GFP-labelled hCECs cultured in 2D monocultures shows high GFP expression.

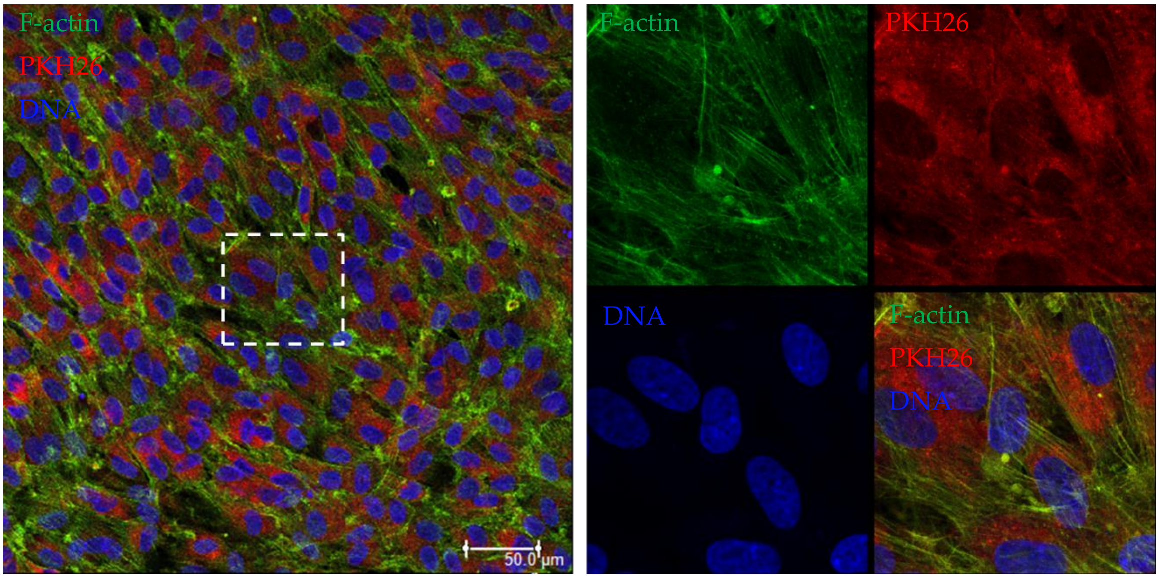

Figure S2. PKH26-stained hCFs show broad cytosolic fluorescent staining. Relatively equal distribution of PKH26 throughout the culture shown in low (*left side*) and high magnification images (*right side*).

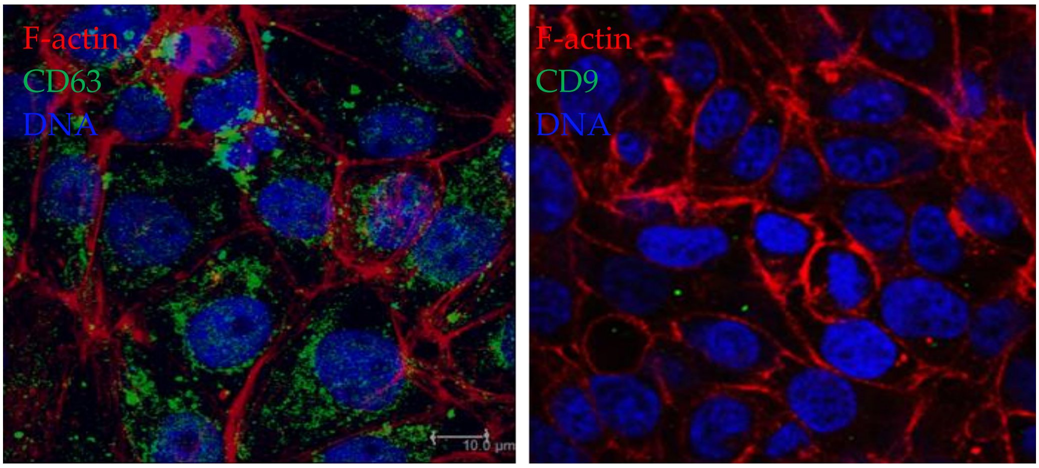

Figure S3. Expression of exosomal markers, CD63 and CD9, in hCECs cultured in 2D monocultures.

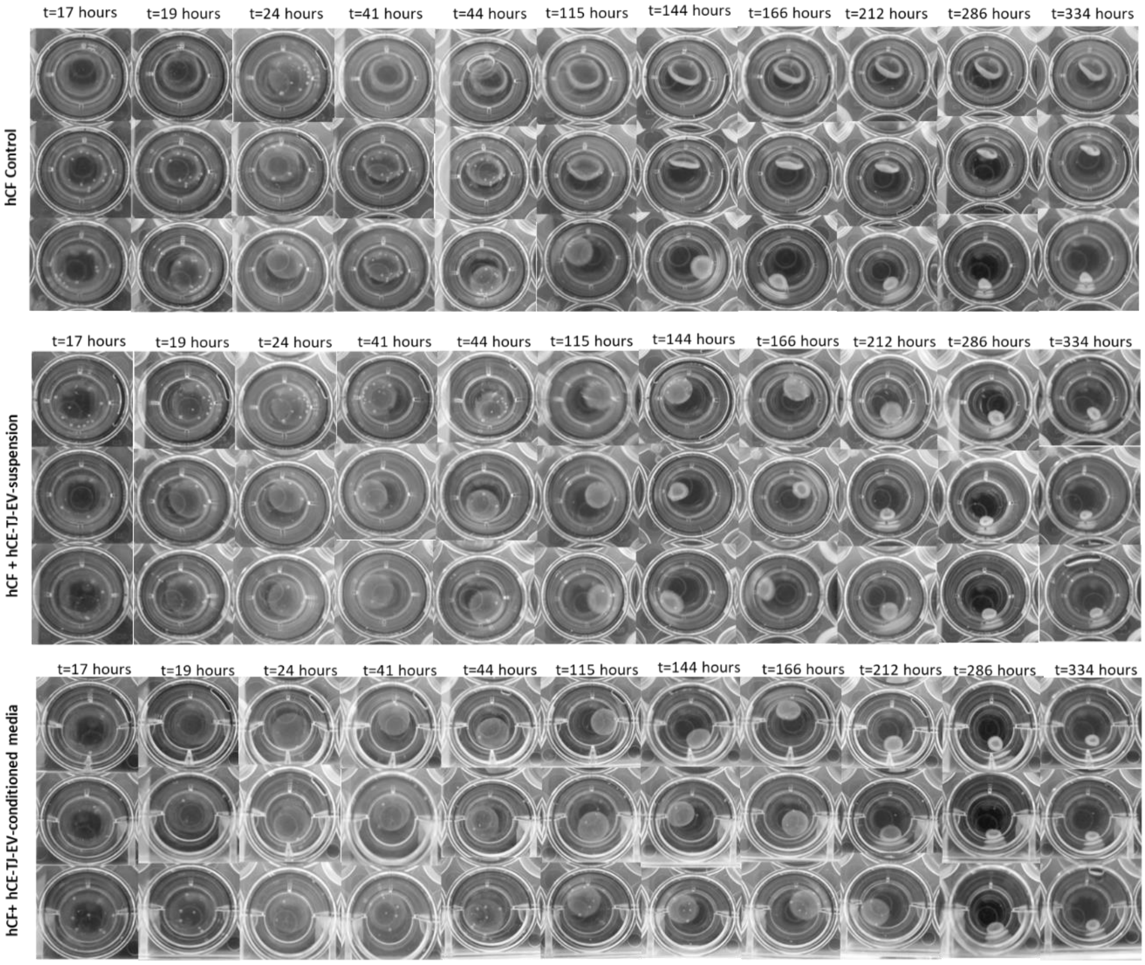

Figure S4. Representative brightfield images of the collagen contraction assay.

Table S1. Proteins identified in isolated epithelial-derived (hCE-TJ) EVs categorized by dominant subcellular origin. Proteins are classified based on nine major subcellular locations: 1) Cytoplasm (Soluble), 2) Cytoplasm (Cytoskeleton/Motor), 3) Cytoplasm (Endosomal Trafficking), 4) Extracellular (Secreted), 5) Plasma Membrane, 6) Mitochondria, 7) Nucleus, 8) ER/Golgi, and 9) Other (Lysosome, Unknown). Classification is based on subcellular localization information described in the the UniProtKB database according to the dominant location based on the epithelial cell type (Ref. Breuza L., *et al.* Database (Oxford). 2016 Feb 20;2016. pii: bav120).

Protein Name	Accession	Localization	# Peptides	MW [kDa]
1-phosphatidylinositol 4,5-bisphosphate phosphodiesterase	Q4KWH8	Cytoplasm (Soluble)	1	189.1
1,4-alpha-glucan-branching enzyme	Q04446	Cytoplasm (Soluble)	6	80.4
3-hydroxybutyrate dehydrogenase type 2	Q9BUT1	Cytoplasm (Soluble)	1	26.7
3-phosphoinositide-dependent protein kinase 1	O15530	Cytoplasm (Soluble)	1	63.1
3'(2'),5'-bisphosphate nucleotidase 1	O95861	Cytoplasm (Soluble)	1	33.4
4-trimethylaminobutyraldehyde dehydrogenase	P49189	Cytoplasm (Soluble)	5	53.8
6-phosphogluconolactonase	O95336	Cytoplasm (Soluble)	2	27.5
6-phosphogluconate dehydrogenase	P52209	Cytoplasm (Soluble)	6	53.1
14-3-3 protein gamma	P61981	Cytoplasm (Soluble)	7	28.3
14-3-3 protein epsilon	P62258	Cytoplasm (Soluble)	3	29.2
14-3-3 protein eta	Q04917	Cytoplasm (Soluble)	5	28.2
14-3-3 protein theta	P27348	Cytoplasm (Soluble)	12	27.7
14-3-3 protein zeta/delta	P63104	Cytoplasm (Soluble)	6	27.7
26S proteasome non-ATPase regulatory subunit 2	Q13200	Cytoplasm (Soluble)	3	100.1
26S proteasome non-ATPase regulatory subunit 3	O43242	Cytoplasm (Soluble)	4	60.9
26S protease regulatory subunit 4	P62191	Cytoplasm (Soluble)	5	49.2
26S protease regulatory subunit 6A	P17980	Cytoplasm (Soluble)	2	49.2
26S protease regulatory subunit 6B	P43686	Cytoplasm (Soluble)	3	47.3
26S protease regulatory subunit 7	P35998	Cytoplasm (Soluble)	5	48.6
26S proteasome non-ATPase regulatory subunit 8	P48556	Cytoplasm (Soluble)	2	39.6
26S protease regulatory subunit 10B	P62333	Cytoplasm (Soluble)	2	44.1
26S proteasome non-ATPase regulatory subunit 11	O00231	Cytoplasm (Soluble)	2	47.4
26S proteasome non-ATPase regulatory subunit 12	O00232	Cytoplasm (Soluble)	4	52.9
26S proteasome non-ATPase regulatory subunit 13	Q9UNM6	Cytoplasm (Soluble)	4	42.9
26S proteasome non-ATPase regulatory subunit 14	O00487	Cytoplasm (Soluble)	1	34.6
40S ribosomal protein S2	P15880	Cytoplasm (Soluble)	8	31.3
40S ribosomal protein SA	P08865	Cytoplasm (Soluble)	3	32.8
40S ribosomal protein S3	P23396	Cytoplasm (Soluble)	12	26.7
40S ribosomal protein S3a	P61247	Cytoplasm (Soluble)	5	29.9
40S ribosomal protein S4, X isoform	P62701	Cytoplasm (Soluble)	9	29.6
40S ribosomal protein S5	P46782	Cytoplasm (Soluble)	2	22.9
40S ribosomal protein S7	P62081	Cytoplasm (Soluble)	1	22.1

40S ribosomal protein S8	P62241	Cytoplasm (Soluble)	1	24.2
40S ribosomal protein S9	P46781	Cytoplasm (Soluble)	6	22.6
40S ribosomal protein S11	P62280	Cytoplasm (Soluble)	3	18.4
40S ribosomal protein S13	P62277	Cytoplasm (Soluble)	5	17.2
40S ribosomal protein S14	P62263	Cytoplasm (Soluble)	1	16.3
40S ribosomal protein S15a	P62244	Cytoplasm (Soluble)	2	14.8
40S ribosomal protein S16	P62249	Cytoplasm (Soluble)	5	16.4
40S ribosomal protein S18	P62269	Cytoplasm (Soluble)	1	17.7
40S ribosomal protein S19	P39019	Cytoplasm (Soluble)	1	16.1
40S ribosomal protein S25	P62851	Cytoplasm (Soluble)	3	13.7
40S ribosomal protein S26	P62854	Cytoplasm (Soluble)	1	13
40S ribosomal protein S27-like	Q71UM5	Cytoplasm (Soluble)	1	9.5
60S ribosomal protein L4	P36578	Cytoplasm (Soluble)	2	47.7
60S ribosomal protein L5	P46777	Cytoplasm (Soluble)	3	34.3
60S ribosomal protein L7	P18124	Cytoplasm (Soluble)	2	29.2
60S ribosomal protein L9	P32969	Cytoplasm (Soluble)	1	21.9
60S ribosomal protein L10a	P62906	Cytoplasm (Soluble)	1	24.8
60S ribosomal protein L11	P62913	Cytoplasm (Soluble)	3	20.2
60S ribosomal protein L12	P30050	Cytoplasm (Soluble)	2	17.8
60S ribosomal protein L13	P26373	Cytoplasm (Soluble)	1	24.2
60S ribosomal protein L22	P35268	Cytoplasm (Soluble)	1	14.8
60S ribosomal protein L23	P62829	Cytoplasm (Soluble)	1	14.9
60S ribosomal protein L30	P62888	Cytoplasm (Soluble)	1	12.8
60S ribosomal protein L34	P49207	Cytoplasm (Soluble)	2	13.3
60S ribosomal protein L35	P42766	Cytoplasm (Soluble)	1	14.5
60S ribosomal protein L36	Q9Y3U8	Cytoplasm (Soluble)	1	12.2
60S acidic ribosomal protein P0	P05388	Cytoplasm (Soluble)	4	34.3
60S acidic ribosomal protein P2	P05387	Cytoplasm (Soluble)	1	11.7
Acyl-protein thioesterase 2	O95372	Cytoplasm (Soluble)	1	24.7
Adenylate kinase isoenzyme 1	P00568	Cytoplasm (Soluble)	2	21.6
Adenosylhomocysteinase	P23526	Cytoplasm (Soluble)	9	47.7
Adenylyl cyclase-associated protein 1	Q01518	Cytoplasm (Soluble)	1	51.9
ADP-ribosylation factor 3	P61204	Cytoplasm (Soluble)	4	20.6
Adenosine kinase	P55263	Cytoplasm (Soluble)	2	40.5
Adenosylhomocysteinase 2	O43865	Cytoplasm (Soluble)	6	58.9
Alanine-tRNA ligase	P49588	Cytoplasm (Soluble)	3	106.7
Alcohol dehydrogenase class-3	P11766	Cytoplasm (Soluble)	5	39.7
Alcohol dehydrogenase	P14550	Cytoplasm (Soluble)	2	36.6
Aldehyde dehydrogenase family 1 member A3	P47895	Cytoplasm (Soluble)	4	56.1
Aldose 1-epimerase	Q96C23	Cytoplasm (Soluble)	1	37.7
Aldose reductase	P15121	Cytoplasm (Soluble)	5	35.8
Alpha-aminoadipic semialdehyde dehydrogenase	P49419	Cytoplasm (Soluble)	5	58.5

Alpha-enolase	P06733	Cytoplasm (Soluble)	8	47.1
Aminoacyl tRNA synthase complex	Q12904	Cytoplasm (Soluble)	1	34.3
AMP deaminase 2	Q01433	Cytoplasm (Soluble)	2	100.6
Asparagine-tRNA ligase	O43776	Cytoplasm (Soluble)	1	62.9
Aspartyl aminopeptidase	Q9ULA0	Cytoplasm (Soluble)	1	52.4
Aspartate aminotransferase	P17174	Cytoplasm (Soluble)	4	46.2
Aspartate-tRNA ligase	P14868	Cytoplasm (Soluble)	2	57.1
ATP-citrate synthase	P53396	Cytoplasm (Soluble)	8	120.8
ATP-dependent RNA helicase DDX1	Q92499	Cytoplasm (Soluble)	3	82.4
Basic leucine zipper and W2 domain-containing protein 1	Q7L1Q6	Cytoplasm (Soluble)	1	48
Bifunctional glutamate/proline--tRNA ligase	P07814	Cytoplasm (Soluble)	3	170.5
Bifunctional purine biosynthesis protein PURH	P31939	Cytoplasm (Soluble)	6	64.6
Bleomycin hydrolase	Q13867	Cytoplasm (Soluble)	1	52.5
C-1-tetrahydrofolate synthase, cytoplasmic	P11586	Cytoplasm (Soluble)	5	101.5
C-terminal-binding protein 1	Q13363	Cytoplasm (Soluble)	2	47.5
Calcyclin-binding protein	Q9HB71	Cytoplasm (Soluble)	1	26.2
Calreticulin	P27797	Cytoplasm (Soluble)	1	48.1
cAMP-dependent protein kinase catalytic subunit beta	P22694	Cytoplasm (Soluble)	1	40.6
cGMP-dependent protein kinase 1	Q13976	Cytoplasm (Soluble)	1	76.3
Coatomer subunit beta'	P35606	Cytoplasm (Soluble)	2	102.4
Complement C3	P01024	Cytoplasm (Soluble)	5	187
Complement factor I	P05156	Cytoplasm (Soluble)	3	65.7
COP9 signalosome complex subunit 1	Q13098	Cytoplasm (Soluble)	3	55.5
COP9 signalosome complex subunit 2	P61201	Cytoplasm (Soluble)	2	51.6
COP9 signalosome complex subunit 4	Q9BT78	Cytoplasm (Soluble)	3	46.2
COP9 signalosome complex subunit 5	Q92905	Cytoplasm (Soluble)	2	37.6
CTP synthase 2	Q9NRF8	Cytoplasm (Soluble)	1	65.6
Cullin-associated NEDD8-dissociated protein 1	Q86VP6	Cytoplasm (Soluble)	11	136.3
Cytoplasm aminopeptidase	P28838	Cytoplasm (Soluble)	2	56.1
D-3-phosphoglycerate dehydrogenase	O43175	Cytoplasm (Soluble)	3	56.6
Dihydropteridine reductase	P09417	Cytoplasm (Soluble)	1	25.8
Dihydropyrimidinase-related protein 2	Q16555	Cytoplasm (Soluble)	11	62.3
Dihydropyrimidinase-related protein 3	Q14195	Cytoplasm (Soluble)	7	61.9
Dihydropyrimidinase-related protein 5	Q9BPU6	Cytoplasm (Soluble)	3	61.4
DNA damage-binding protein 1	Q16531	Cytoplasm (Soluble)	5	126.9
DnaJ homolog subfamily B member 1	P25685	Cytoplasm (Soluble)	1	38
E3 ubiquitin-protein ligase HUWE1	Q7Z6Z7	Cytoplasm (Soluble)	2	481.6
E3 ubiquitin-protein ligase RNF213	Q63HN8	Cytoplasm (Soluble)	1	591
Elongation factor 1-alpha 1	P68104	Cytoplasm (Soluble)	12	50.1
Elongation factor 1-gamma	P26641	Cytoplasm (Soluble)	9	50.1

Elongation factor 2	P13639	Cytoplasm (Soluble)	19	95.3
Eukaryotic initiation factor 4A-II	Q14240	Cytoplasm (Soluble)	5	46.4
Eukaryotic peptide chain release factor	P15170	Cytoplasm (Soluble)	2	55.7
Eukaryotic translation initiation factor 2 subunit 1	P05198	Cytoplasm (Soluble)	4	36.1
Eukaryotic translation initiation factor 2 subunit 3	P41091	Cytoplasm (Soluble)	2	51.1
Eukaryotic translation initiation factor 3	B5ME19	Cytoplasm (Soluble)	3	105.4
Eukaryotic translation initiation factor 3 subunit A	Q14152	Cytoplasm (Soluble)	1	166.5
Eukaryotic translation initiation factor 3 subunit D	O15371	Cytoplasm (Soluble)	1	63.9
Eukaryotic translation initiation factor 3 subunit F	O00303	Cytoplasm (Soluble)	2	37.5
Eukaryotic translation initiation factor 3 subunit L	Q9Y262	Cytoplasm (Soluble)	6	66.7
Eukaryotic translation initiation factor 3 subunit H	O15372	Cytoplasm (Soluble)	1	39.9
Eukaryotic translation initiation factor 3 subunit I	Q13347	Cytoplasm (Soluble)	2	36.5
Eukaryotic initiation factor 4A-I	P60842	Cytoplasm (Soluble)	4	46.1
Eukaryotic translation initiation factor 5A-2	Q9GZV4	Cytoplasm (Soluble)	2	16.8
Fatty acid synthase	P49327	Cytoplasm (Soluble)	22	273.3
Fermitin family homolog 2	Q96AC1	Cytoplasm (Soluble)	1	77.8
Ferritin heavy chain	P02794	Cytoplasm (Soluble)	2	21.2
Fructose-bisphosphate aldolase A	P04075	Cytoplasm (Soluble)	8	39.4
General vesicular transport factor p115	O60763	Cytoplasm (Soluble)	2	107.8
Glucose-6-phosphate 1-dehydrogenase	P11413	Cytoplasm (Soluble)	3	59.2
Glutathione S-transferase Mu 3	P21266	Cytoplasm (Soluble)	3	26.5
Glutathione S-transferase P	P09211	Cytoplasm (Soluble)	1	23.3
Glycine--tRNA ligase	P41250	Cytoplasm (Soluble)	5	83.1
Glyceraldehyde-3-phosphate dehydrogenase	P04406	Cytoplasm (Soluble)	12	36
Glycylpeptide N-tetradecanoyltransferase 1	P30419	Cytoplasm (Soluble)	1	56.8
Glycogen debranching enzyme	P35573	Cytoplasm (Soluble)	2	174.7
Glycogen phosphorylase	P06737	Cytoplasm (Soluble)	6	97.1
GMP reductase 2	Q9P2T1	Cytoplasm (Soluble)	4	37.9
GTPase KRas	P01116	Cytoplasm (Soluble)	1	21.6
GTP-binding nuclear protein Ran	P62826	Cytoplasm (Soluble)	2	24.4
Guanine nucleotide-binding protein	P62873	Cytoplasm (Soluble)	2	37.4
Guanine nucleotide-binding protein	P62879	Cytoplasm (Soluble)	2	37.3
Isocitrate dehydrogenase [NADP] cytoplasmic	O75874	Cytoplasm (Soluble)	6	46.6
Heat shock 70 kDa protein 1B	P0DMV9	Cytoplasm (Soluble)	11	70
Heat shock-related 70 kDa protein 2	P54652	Cytoplasm (Soluble)	8	70
Heat shock 70 kDa protein 4	P34932	Cytoplasm (Soluble)	11	94.3
Heat shock protein beta-1	P04792	Cytoplasm (Soluble)	3	22.8
Heat shock protein HSP 90-alpha	P07900	Cytoplasm (Soluble)	22	84.6
Heat shock protein HSP 90-beta	P08238	Cytoplasm (Soluble)	18	83.2
Heat shock cognate 71 kDa protein	P11142	Cytoplasm (Soluble)	21	70.9
Hemoglobin subunit alpha	P69905	Cytoplasm (Soluble)	4	15.2
Hemoglobin subunit beta	P68871	Cytoplasm (Soluble)	3	16

Hemoglobin subunit gamma-1	P69891	Cytoplasm (Soluble)	3	16.1
Heterogeneous nuclear ribonucleoprotein A1-like 2	Q32P51	Cytoplasm (Soluble)	1	34.2
Heterogeneous nuclear ribonucleoprotein D0	Q14103	Cytoplasm (Soluble)	1	38.4
Heterogeneous nuclear ribonucleoprotein L	P14866	Cytoplasm (Soluble)	1	64.1
Hexokinase-1	P19367	Cytoplasm (Soluble)	5	102.4
Histidine triad nucleotide-binding protein 1	P49773	Cytoplasm (Soluble)	1	13.8
Isoleucine-tRNA ligase	P41252	Cytoplasm (Soluble)	3	144.4
Inorganic pyrophosphatase	Q15181	Cytoplasm (Soluble)	1	32.6
Inosine triphosphate pyrophosphatase	Q9BY32	Cytoplasm (Soluble)	1	21.4
Ketimine reductase mu-crystallin	Q14894	Cytoplasm (Soluble)	2	33.8
L-lactate dehydrogenase B chain	P07195	Cytoplasm (Soluble)	8	36.6
Lactoylglutathione lyase	Q04760	Cytoplasm (Soluble)	3	20.8
Leukotriene A-4 hydrolase	P09960	Cytoplasm (Soluble)	4	69.2
Lysine-tRNA ligase	Q15046	Cytoplasm (Soluble)	1	68
Major vault protein	Q14764	Cytoplasm (Soluble)	6	99.3
Mannose-1-phosphate guanyltransferase alpha	Q96IJ6	Cytoplasm (Soluble)	1	46.3
Mannose-1-phosphate guanyltransferase beta	Q9Y5P6	Cytoplasm (Soluble)	6	39.8
Methylosome protein 50	Q9BQA1	Cytoplasm (Soluble)	1	36.7
Mitogen-activated protein kinase 1	P28482	Cytoplasm (Soluble)	3	41.4
Mitogen-activated protein kinase 3	P27361	Cytoplasm (Soluble)	5	43.1
Multifunctional protein ADE2	P22234	Cytoplasm (Soluble)	5	47
N-acetylgalactosamine kinase	Q01415	Cytoplasm (Soluble)	1	50.3
N-acylneuraminate cytidylyltransferase	Q8NFW8	Cytoplasm (Soluble)	2	48.3
N-alpha-acetyltransferase 15	Q9BXJ9	Cytoplasm (Soluble)	4	101.2
Neurobeachin	Q8NFP9	Cytoplasm (Soluble)	1	327.6
Neuroendocrine convertase 2	P16519	Cytoplasm (Soluble)	2	70.5
Peptidyl-prolyl cis-trans isomerase A	P62937	Cytoplasm (Soluble)	6	18
Peroxiredoxin-1	Q06830	Cytoplasm (Soluble)	5	22.1
Peroxiredoxin-2	P32119	Cytoplasm (Soluble)	3	21.9
Phenylalanine-tRNA ligase alpha subunit	Q9Y285	Cytoplasm (Soluble)	1	57.5
Phosphoacetylglucosamine mutase	O95394	Cytoplasm (Soluble)	2	59.8
Phosphatidylethanolamine-binding protein 1	P30086	Cytoplasm (Soluble)	1	21
Phosphoglucomutase-1	P36871	Cytoplasm (Soluble)	13	61.4
Phosphoglucomutase-2	Q96G03	Cytoplasm (Soluble)	8	68.2
Phosphoglycerate kinase 1	P00558	Cytoplasm (Soluble)	8	44.6
Phosphoglycerate mutase 1	P18669	Cytoplasm (Soluble)	5	28.8
Phosphoribosyl pyrophosphate synthase-associated protein 1	Q14558	Cytoplasm (Soluble)	1	39.4
Platelet-activating factor acetylhydrolase IB	Q15102	Cytoplasm (Soluble)	3	25.7
Poly(rC)-binding protein 4	P57723	Cytoplasm (Soluble)	1	41.5
Pre-mRNA-processing factor 19	Q9UMS4	Cytoplasm (Soluble)	3	55.1
Programmed cell death 6-interacting protein	Q8WUM4	Cytoplasm (Soluble)	9	96

Proliferation-associated protein 2G4	Q9UQ80	Cytoplasm (Soluble)	1	43.8
Prostaglandin E synthase 3	Q15185	Cytoplasm (Soluble)	1	18.7
Proteasome subunit alpha type-1	P25786	Cytoplasm (Soluble)	3	29.5
Proteasome subunit alpha type-2	P25787	Cytoplasm (Soluble)	5	25.9
Proteasome subunit alpha type-3	P25788	Cytoplasm (Soluble)	3	28.4
Proteasome subunit alpha type-4	P25789	Cytoplasm (Soluble)	3	29.5
Proteasome subunit alpha type-6	P60900	Cytoplasm (Soluble)	4	27.4
Proteasome subunit alpha type-7	O14818	Cytoplasm (Soluble)	4	27.9
Proteasome subunit beta type-2	P49721	Cytoplasm (Soluble)	2	22.8
Proteasome subunit beta type-3	P49720	Cytoplasm (Soluble)	1	22.9
Proteasome subunit beta type-4	P28070	Cytoplasm (Soluble)	2	29.2
Proteasome subunit beta type-5	P28074	Cytoplasm (Soluble)	1	28.5
Proteasome subunit beta type-6	P28072	Cytoplasm (Soluble)	2	25.3
Proteasome subunit beta type-7	Q99436	Cytoplasm (Soluble)	3	29.9
Protein arginine N-methyltransferase 1	Q99873	Cytoplasm (Soluble)	3	41.5
Protein deglycase DJ-1	Q99497	Cytoplasm (Soluble)	2	19.9
Protein NDRG2	Q9UN36	Cytoplasm (Soluble)	2	40.8
Puromycin-sensitive aminopeptidase	P55786	Cytoplasm (Soluble)	17	103.2
Pyrroline-5-carboxylate reductase 3	Q53H96	Cytoplasm (Soluble)	1	28.6
Pyruvate kinase PKM	P14618	Cytoplasm (Soluble)	21	57.9
Quinone oxidoreductase	Q08257	Cytoplasm (Soluble)	2	35.2
Rab GDP dissociation inhibitor alpha	P31150	Cytoplasm (Soluble)	5	50.6
Ran-specific GTPase-activating protein	P43487	Cytoplasm (Soluble)	1	23.3
Receptor of activated protein C kinase 1	P63244	Cytoplasm (Soluble)	6	35.1
Retinal dehydrogenase 1	P00352	Cytoplasm (Soluble)	8	54.8
Retinal dehydrogenase 2	O94788	Cytoplasm (Soluble)	6	56.7
Ribosome maturation protein SBDS	Q9Y3A5	Cytoplasm (Soluble)	1	28.7
Ribose-phosphate pyrophosphokinase 2	P11908	Cytoplasm (Soluble)	2	34.7
S-adenosylmethionine synthase isoform type-2	P31153	Cytoplasm (Soluble)	2	43.6
S-formylglutathione hydrolase	P10768	Cytoplasm (Soluble)	3	31.4
Secernin-1	Q12765	Cytoplasm (Soluble)	1	46.4
Selenium-binding protein 1	Q13228	Cytoplasm (Soluble)	2	52.4
Serine/threonine-protein kinase PAK 2	Q13177	Cytoplasm (Soluble)	1	58
Serine/threonine-protein phosphatase 2A	P67775	Cytoplasm (Soluble)	4	35.6
Serine/threonine-protein phosphatase 2A	P30153	Cytoplasm (Soluble)	5	65.3
Serine hydroxymethyltransferase	P34897	Cytoplasm (Soluble)	6	56
Serine-threonine kinase receptor-associated protein	Q9Y3F4	Cytoplasm (Soluble)	2	38.4
Serine-tRNA ligase	P49591	Cytoplasm (Soluble)	1	58.7
Sialic acid synthase	Q9NR45	Cytoplasm (Soluble)	4	40.3
Spermidine synthase	P19623	Cytoplasm (Soluble)	1	33.8
Spermine synthase	P52788	Cytoplasm (Soluble)	2	41.2
SUMO-activating enzyme subunit 1	Q9UBE0	Cytoplasm (Soluble)	1	38.4

SUMO-activating enzyme subunit 2	Q9UBT2	Cytoplasm (Soluble)	5	71.2
Superoxide dismutase	P00441	Cytoplasm (Soluble)	1	15.9
T-complex protein 1 subunit alpha	P17987	Cytoplasm (Soluble)	5	60.3
T-complex protein 1 subunit beta	P78371	Cytoplasm (Soluble)	10	57.5
T-complex protein 1 subunit gamma	P49368	Cytoplasm (Soluble)	7	60.5
T-complex protein 1 subunit zeta	P40227	Cytoplasm (Soluble)	7	58
T-complex protein 1 subunit eta	Q99832	Cytoplasm (Soluble)	7	59.3
T-complex protein 1 subunit theta	P50990	Cytoplasm (Soluble)	13	59.6
Threonine--tRNA ligase, cytoplasmic	P26639	Cytoplasm (Soluble)	3	83.4
Thioredoxin-like protein 1	O43396	Cytoplasm (Soluble)	1	32.2
Transgelin	Q01995	Cytoplasm (Soluble)	1	22.6
Transgelin-2	P37802	Cytoplasm (Soluble)	1	22.4
Transitional endoplasmic reticulum ATPase	P55072	Cytoplasm (Soluble)	17	89.3
Transketolase	P29401	Cytoplasm (Soluble)	9	67.8
Triosephosphate isomerase	P60174	Cytoplasm (Soluble)	6	30.8
Tripeptidyl-peptidase 2	P29144	Cytoplasm (Soluble)	2	138.3
Triple functional domain protein	O75962	Cytoplasm (Soluble)	1	346.7
tRNA-splicing ligase RtcB homolog	Q9Y3I0	Cytoplasm (Soluble)	1	55.2
Tryptophan--tRNA ligase, cytoplasmic	P23381	Cytoplasm (Soluble)	1	53.1
Ubiquitin-40S ribosomal protein S27a	P62979	Cytoplasm (Soluble)	6	18
Ubiquitin-like modifier-activating enzyme 1	P22314	Cytoplasm (Soluble)	10	117.8
Ubiquitin carboxyl-terminal hydrolase 7	Q93009	Cytoplasm (Soluble)	1	128.2
UDP-N-acetylhexosamine pyrophosphorylase	Q16222	Cytoplasm (Soluble)	1	58.7
UMP-CMP kinase	P30085	Cytoplasm (Soluble)	1	22.2
UTP-glucose-1-phosphate uridylyltransferase	Q16851	Cytoplasm (Soluble)	3	56.9
V-type proton ATPase catalytic subunit A	P38606	Cytoplasm (Soluble)	5	68.3
V-type proton ATPase subunit B	P21281	Cytoplasm (Soluble)	6	56.5
V-type proton ATPase subunit H	Q9UI12	Cytoplasm (Soluble)	1	55.8
Valine-tRNA ligase	P26640	Cytoplasm (Soluble)	2	140.4
WD repeat-containing protein 1	O75083	Cytoplasm (Soluble)	9	66.2
WD repeat-containing protein 61	Q9GZS3	Cytoplasm (Soluble)	1	33.6
Actin, aortic smooth muscle	P62736	Cytoplasm (Cytoskeleton/Motor)	9	42
Actin, cytoplasmic 1	P60709	Cytoplasm (Cytoskeleton/Motor)	15	41.7
Actin-related protein 2/3 complex subunit 1A	Q92747	Cytoplasm (Cytoskeleton/Motor)	5	41.5
Actin-related protein 2	P61160	Cytoplasm (Cytoskeleton/Motor)	5	44.7
Actin-related protein 2/3 complex subunit 2	O15144	Cytoplasm (Cytoskeleton/Motor)	1	34.3

Actin-related protein 2/3 complex subunit 3	O15145	Cytoplasm (Cytoskeleton/Motor)	4	20.5
Actin-related protein 2/3 complex subunit 4	P59998	Cytoplasm (Cytoskeleton/Motor)	4	19.7
Actin-related protein 3	P61158	Cytoplasm (Cytoskeleton/Motor)	5	47.3
Actin-like protein 6B	O94805	Cytoplasm (Cytoskeleton/Motor)	1	46.8
ADP-ribosylation factor-like protein 3	P36405	Cytoplasm (Cytoskeleton/Motor)	2	20.4
Alpha-actinin-3	Q08043	Cytoplasm (Cytoskeleton/Motor)	2	103.2
Alpha-centractin	P61163	Cytoplasm (Cytoskeleton/Motor)	4	42.6
Band 4.1-like protein 2	O43491	Cytoplasm (Cytoskeleton/Motor)	4	112.5
Cadherin-1	P12830	Cytoplasm (Cytoskeleton/Motor)	1	97.4
Cell division control protein 42 homolog	P60953	Cytoplasm (Cytoskeleton/Motor)	2	21.2
Cofilin-1	P23528	Cytoplasm (Cytoskeleton/Motor)	3	18.5
Coronin-1A	P31146	Cytoplasm (Cytoskeleton/Motor)	1	51
Coronin-1B	Q9BR76	Cytoplasm (Cytoskeleton/Motor)	3	54.2
Cytoplasmic dynein 1 heavy chain 1	Q14204	Cytoplasm (Cytoskeleton/Motor)	2	532.1
Dynamin-1-like protein	O00429	Cytoplasm (Cytoskeleton/Motor)	2	81.8
Echinoderm microtubule-associated protein-like 2	O95834	Cytoplasm (Cytoskeleton/Motor)	1	70.6
Ezrin	P15311	Cytoplasm (Cytoskeleton/Motor)	6	69.4
Fascin	Q16658	Cytoplasm (Cytoskeleton/Motor)	4	54.5
Filamin-A	P21333	Cytoplasm (Cytoskeleton/Motor)	25	280.6
Filamin-B	O75369	Cytoplasm (Cytoskeleton/Motor)	10	278
Filamin-C	Q14315	Cytoplasm (Cytoskeleton/Motor)	4	290.8

Heterogeneous nuclear ribonucleoprotein U	Q00839	Cytoplasm (Cytoskeleton/Motor)	3	90.5
Keratin, type I cytoskeletal 14	P02533	Cytoplasm (Cytoskeleton/Motor)	4	51.5
Kinesin-like protein KIF21A	Q7Z4S6	Cytoplasm (Cytoskeleton/Motor)	1	187.1
Microtubule-associated protein 1B	P46821	Cytoplasm (Cytoskeleton/Motor)	1	270.5
Mitogen-activated protein kinase kinase 1	Q02750	Cytoplasm (Cytoskeleton/Motor)	1	43.4
Myosin-9	P35579	Cytoplasm (Cytoskeleton/Motor)	5	226.4
Myosin-binding protein C, fast-type	Q14324	Cytoplasm (Cytoskeleton/Motor)	1	128
Myosin light chain kinase, smooth muscle	Q15746	Cytoplasm (Cytoskeleton/Motor)	2	210.6
Myosin light polypeptide 6	P60660	Cytoplasm (Cytoskeleton/Motor)	2	16.9
Neurofilament light polypeptide	P07196	Cytoplasm (Cytoskeleton/Motor)	1	61.5
Phosphoglucomutase-like protein 5	Q15124	Cytoplasm (Cytoskeleton/Motor)	1	62.2
Platelet-activating factor acetylhydrolase IB subunit alpha	P43034	Cytoplasm (Cytoskeleton/Motor)	5	46.6
Profilin-1	P07737	Cytoplasm (Cytoskeleton/Motor)	2	15
Profilin-2	P35080	Cytoplasm (Cytoskeleton/Motor)	2	15
Protein farnesyltransferase	P49354	Cytoplasm (Cytoskeleton/Motor)	1	44.4
Septin-2	Q15019	Cytoplasm (Cytoskeleton/Motor)	3	41.5
Septin-7	Q16181	Cytoplasm (Cytoskeleton/Motor)	3	50.6
Septin-9	Q9UHD8	Cytoplasm (Cytoskeleton/Motor)	3	65.4
Septin-10	Q9P0V9	Cytoplasm (Cytoskeleton/Motor)	1	52.6
Septin-11	Q9NVA2	Cytoplasm (Cytoskeleton/Motor)	2	49.4
Talin-1	Q9Y490	Cytoplasm (Cytoskeleton/Motor)	3	269.6

T-complex protein 1 subunit delta	P50991	Cytoplasm (Cytoskeleton/Motor)	8	57.9
Tubulin alpha-1A chain	Q71U36	Cytoplasm (Cytoskeleton/Motor)	5	50.1
Tubulin beta chain	P07437	Cytoplasm (Cytoskeleton/Motor)	8	49.6
Tubulin--tyrosine ligase-like protein 12	Q14166	Cytoplasm (Cytoskeleton/Motor)	1	74.4
T-complex protein 1 subunit epsilon	P48643	Cytoplasm (Cytoskeleton/Motor)	4	59.6
Transforming protein RhoA	P61586	Cytoplasm (Cytoskeleton/Motor)	3	21.8
Tropomyosin alpha-1 chain	P09493	Cytoplasm (Cytoskeleton/Motor)	1	32.7
Tropomodulin-2	Q9NZR1	Cytoplasm (Cytoskeleton/Motor)	1	39.6
Annexin A2	P07355	Cytoplasm (Endosomal Trafficking)	9	38.6
AP-1 complex subunit beta-1	Q10567	Cytoplasm (Endosomal Trafficking)	1	104.6
Calcium-dependent secretion activator 1	Q9ULU8	Cytoplasm (Endosomal Trafficking)	3	152.7
CD9 antigen	P21926	Cytoplasm (Endosomal Trafficking)	1	25.4
Clathrin heavy chain 1	Q00610	Cytoplasm (Endosomal Trafficking)	19	191.5
Clathrin light chain A	P09496	Cytoplasm (Endosomal Trafficking)	5	27.1
Clathrin light chain B	P09497	Cytoplasm (Endosomal Trafficking)	2	25.2
DCC-interacting protein 13-alpha	Q9UKG1	Cytoplasm (Endosomal Trafficking)	1	79.6
Exportin-2	P55060	Cytoplasm (Endosomal Trafficking)	3	110.3
Ras-related protein Rab-1A	P62820	Cytoplasm (Endosomal Trafficking)	5	22.7
Ras-related protein Rap-1b	P61224	Cytoplasm (Endosomal Trafficking)	3	20.8
Ras-related protein Rab-2A	P61019	Cytoplasm (Endosomal Trafficking)	2	23.5
Ras-related protein Rab-6A	P20340	Cytoplasm (Endosomal Trafficking)	3	23.6

Ras-related protein Rab-7a	P51149	Cytoplasm (Endosomal Trafficking)	2	23.5
Ras-related protein Rab-8B	Q92930	Cytoplasm (Endosomal Trafficking)	5	23.6
Ras-related protein Rab-11A	P62491	Cytoplasm (Endosomal Trafficking)	4	24.4
Ras-related protein Rab-14	P61106	Cytoplasm (Endosomal Trafficking)	5	23.9
Ras-related protein Rab-3A	P20336	Cytoplasm (Endosomal Trafficking)	8	25
Ras-related protein Rab-5A	P20339	Cytoplasm (Endosomal Trafficking)	3	23.6
Ras-related protein R-Ras2	P62070	Cytoplasm (Endosomal Trafficking)	1	23.4
Synaptic vesicle membrane protein VAT-1 homolog	Q99536	Cytoplasm (Endosomal Trafficking)	3	41.9
Synaptic vesicle membrane protein VAT-1 homolog-like	Q9HCJ6	Cytoplasm (Endosomal Trafficking)	1	45.9
Vacuolar protein sorting-associated protein 35	Q96QK1	Cytoplasm (Endosomal Trafficking)	3	91.6
Vacuolar protein sorting-associated protein 29	Q9UBQ0	Cytoplasm (Endosomal Trafficking)	2	20.5
Ras-related GTP-binding protein C	Q9HB90	Cytoplasm (Endosomal Trafficking)	1	44.2
Ras-related protein Ral-A	P11233	Cytoplasm (Endosomal Trafficking)	2	23.6
Syntaxin-binding protein 1	P61764	Cytoplasm (Endosomal Trafficking)	4	67.5
Ras-related protein Rab-1B	Q9H0U4	Cytoplasm (Endosomal Trafficking)	5	22.2
Rab GDP dissociation inhibitor beta	P50395	Cytoplasm (Endosomal Trafficking)	4	50.6
Agrin	O00468	Extracellular (Secreted)	14	217.1
Alpha-2-macroglobulin	P01023	Extracellular (Secreted)	1	163.2
Antithrombin-III	P01008	Extracellular (Secreted)	1	52.6
Carboxypeptidase B2	Q96IY4	Extracellular (Secreted)	1	48.4
Collagen alpha-1(VI) chain	P12109	Extracellular (Secreted)	1	108.5
Collagen alpha-2(VI) chain	P12110	Extracellular (Secreted)	1	108.5
Collagen alpha-3(VI) chain	P12111	Extracellular (Secreted)	1	343.5
Complement C1q tumor necrosis factor-related protein 5	Q9BXJ0	Extracellular (Secreted)	1	25.3
Deleted in malignant brain tumors 1 protein	Q9UGM3	Extracellular (Secreted)	2	260.6

Endoplasmin	P14625	Extracellular (Secreted)	23	92.4
Endothelial lipase	Q9Y5X9	Extracellular (Secreted)	1	56.8
Epithelial cell adhesion molecule	P16422	Extracellular (Secreted)	1	34.9
Fibromodulin	Q06828	Extracellular (Secreted)	1	43.2
Fibronectin	P02751	Extracellular (Secreted)	58	262.5
Galectin-1	P09382	Extracellular (Secreted)	1	14.7
Galectin-3-binding protein	Q08380	Extracellular (Secreted)	13	65.3
Glia-derived nexin	P07093	Extracellular (Secreted)	1	44
Glycogen phosphorylase, brain form	P11216	Extracellular (Secreted)	2	96.6
Growth hormone variant	P01242	Extracellular (Secreted)	1	25
Haloacid dehalogenase-like	Q9H0R4	Extracellular (Secreted)	2	28.5
Hemopexin	P02790	Extracellular (Secreted)	8	51.6
Ig alpha-1 chain C region	P01876	Extracellular (Secreted)	1	37.6
Ig gamma-1 chain C region	P01857	Extracellular (Secreted)	5	36.1
Ig gamma-2 chain C region	P01859	Extracellular (Secreted)	4	35.9
Insulin	P01308	Extracellular (Secreted)	1	12
Insulin-like growth factor-binding protein 3	P17936	Extracellular (Secreted)	1	31.7
Inter-alpha-trypsin inhibitor heavy chain H4	Q14624	Extracellular (Secreted)	3	103.3
Keratin, type I cytoskeletal 10	P13645	Extracellular (Secreted)	14	58.8
Lactadherin	Q08431	Extracellular (Secreted)	3	43.1
Laminin subunit alpha-3	Q16787	Extracellular (Secreted)	8	366.4
Laminin subunit alpha-4	Q16363	Extracellular (Secreted)	1	202.4
Laminin subunit beta-1	P07942	Extracellular (Secreted)	9	197.9
Laminin subunit gamma-1	P11047	Extracellular (Secreted)	5	177.5
Laminin subunit gamma-2	Q13753	Extracellular (Secreted)	1	130.9
Probable phosphoglycerate mutase 4	Q8N0Y7	Extracellular (Secreted)	2	28.8
Prolargin	P51888	Extracellular (Secreted)	1	43.8
Protein CYR61	O00622	Extracellular (Secreted)	1	42
Protein S100-A4	P26447	Extracellular (Secreted)	1	11.7
Secretogranin-3	Q8WXD2	Extracellular (Secreted)	1	53
Serotransferrin	P02787	Extracellular (Secreted)	20	77
Sushi repeat-containing protein SRPX2	O60687	Extracellular (Secreted)	1	52.9
Thioredoxin	P10599	Extracellular (Secreted)	1	11.7
Thrombospondin-1	P07996	Extracellular (Secreted)	36	129.3
Transforming growth factor-beta-induced protein ig-h3	Q15582	Extracellular (Secreted)	6	74.6
Trypsin-1	P07477	Extracellular (Secreted)	1	26.5
4F2 cell-surface antigen heavy chain	P08195	Plasma Membrane	4	68
5'-nucleotidase	P21589	Plasma Membrane (GPI-anchored)	1	63.3
AP-2 complex subunit beta	P63010	Plasma Membrane	3	104.5
AP-2 complex subunit mu	Q96CW1	Plasma Membrane	1	49.6

AP-2 complex subunit sigma	P53680	Plasma Membrane	1	17
Basigin	P35613	Plasma Membrane	1	42.2
Calpain-2 catalytic subunit	P17655	Plasma Membrane	1	79.9
cAMP-dependent protein kinase	P31323	Plasma Membrane	2	46.3
CD44 antigen	P16070	Plasma Membrane	1	81.5
Chloride intracellular channel protein 1	O00299	Plasma Membrane	2	26.9
Ciliogenesis and planar polarity effector 1	Q9H799	Plasma Membrane	1	361.5
Facilitated glucose transporter member 1	P11166	Plasma Membrane	2	54
Gamma-enolase	P09104	Plasma Membrane	5	47.2
Guanine nucleotide-binding protein G(o) subunit alpha	P09471	Plasma Membrane	2	40
Guanine nucleotide-binding protein G(s) subunit alpha	Q5JWF2	Plasma Membrane	2	111
Ig lambda-2 chain C regions	P0CG05	Plasma Membrane	1	11.3
Ig kappa chain V-I region AG	P01593	Plasma Membrane	1	12
Immunoglobulin superfamily member 1	Q8N6C5	Plasma Membrane	4	148.8
Immunoglobulin superfamily member 8	Q969P0	Plasma Membrane	3	65
Integrin alpha-3	P26006	Plasma Membrane	1	116.5
Integrin beta-1	P05556	Plasma Membrane	4	88.4
LanC-like protein 1	O43813	Plasma Membrane	2	45.3
Large neutral amino acids transporter	Q01650	Plasma Membrane	2	55
Protein disulfide-isomerase	P07237	Plasma Membrane (Peripheral)	1	57.1
Ras-related C3 botulinum toxin substrate 1	P63000	Plasma Membrane	3	21.4
Retinoic acid-induced protein 3	Q8NFJ5	Plasma Membrane	1	40.2
Rho guanine nucleotide exchange factor 18	Q6ZSZ5	Plasma Membrane	1	130.7
Sodium/potassium-transporting ATPase subunit alpha-1	P05023	Plasma Membrane	6	112.8
Tyrosine-protein kinase Lck	P06239	Plasma Membrane	1	58
V-type proton ATPase subunit d 1	P61421	Plasma Membrane	1	40.3
2-oxoglutarate dehydrogenase-like	Q9ULD0	Mitochondria	1	114.4
10 kDa heat shock protein	P61604	Mitochondria	1	10.9
60 kDa heat shock protein	P10809	Mitochondria	8	61
Aconitate hydratase	Q99798	Mitochondria	5	85.4
Aldehyde dehydrogenase	P05091	Mitochondria	5	56.3
Aspartate aminotransferase	P00505	Mitochondria	5	47.5
ATP synthase subunit alpha	P25705	Mitochondria	6	59.7
ATP synthase subunit beta	P06576	Mitochondria	8	56.5
Citrate synthase	O75390	Mitochondria	4	51.7
Dihydrolipoyl dehydrogenase	P09622	Mitochondria	1	54.1
Dihydrolipoyllysine-residue acetyltransferase	P10515	Mitochondria	4	69
Enoyl-CoA hydratase	P30084	Mitochondria	1	31.4

Fumarate hydratase	P07954	Mitochondria	1	54.6
Glutamate dehydrogenase 1	P00367	Mitochondria	1	61.4
Glutathione reductase	P00390	Mitochondria	1	56.2
Histidine triad nucleotide-binding protein 2	Q9BX68	Mitochondria	1	17.2
Hydroxyacylglutathione hydrolase	Q16775	Mitochondria	1	33.8
Isovaleryl-CoA dehydrogenase	P26440	Mitochondria	2	46.3
Leucine-rich PPR motif-containing protein	P42704	Mitochondria	1	157.8
Malate dehydrogenase	P40926	Mitochondria	6	35.5
Methylmalonyl-CoA mutase	P22033	Mitochondria	1	83.1
Mitochondrial-processing peptidase subunit beta	O75439	Mitochondria	1	54.3
Mitochondrial 10-formyltetrahydrofolate dehydrogenase	Q3SY69	Mitochondria	2	101.7
NAD(P)H-hydrate epimerase	Q8NCW5	Mitochondria	1	31.7
Oxygen-dependent coproporphyrinogen-III oxidase	P36551	Mitochondria	1	50.1
Peroxiredoxin-5	P30044	Mitochondria	2	22.1
Pyruvate dehydrogenase E1 component subunit alpha	P08559	Mitochondria	2	43.3
Pyruvate dehydrogenase E1 component subunit beta	P11177	Mitochondria	1	39.2
Single-stranded DNA-binding protein	Q04837	Mitochondria	1	17.2
Stress-70 protein	P38646	Mitochondria	1	73.6
Succinate-semialdehyde dehydrogenase	P51649	Mitochondria	1	57.2
Succinyl-CoA:3-ketoacid coenzyme A transferase 1	P55809	Mitochondria	2	56.1
Succinyl-CoA ligase [ADP/GDP-forming] subunit alpha	P53597	Mitochondria	2	36.2
Succinyl-CoA ligase [ADP-forming] subunit beta	Q9P2R7	Mitochondria	1	50.3
Trifunctional enzyme subunit alpha	P40939	Mitochondria	2	82.9
Thiosulfate sulfurtransferase	Q16762	Mitochondria	3	33.4
Very long-chain specific acyl-CoA dehydrogenase	P49748	Mitochondria	1	70.3
5'-3' exoribonuclease 2	Q9H0D6	Nucleus	1	108.5
ATP-dependent RNA helicase DDX3X	O00571	Nucleus	1	73.2
Beta-catenin-like protein 1	Q8WYA6	Nucleus	1	65.1
Bloom syndrome protein	P54132	Nucleus	1	158.9
Cystathionine beta-synthase-like protein	P0DN79	Nucleus	1	60.5
Cullin-4B	Q13620	Nucleus	10	103.9
Elongation factor 1-alpha 2	Q05639	Nucleus	10	50.4
Elongation factor 1-delta	P29692	Nucleus	1	31.1
Flap endonuclease 1	P39748	Nucleus	1	42.6
Guanine nucleotide-binding protein G(q) subunit alpha	P50148	Nucleus	1	42.1
Heterogeneous nuclear ribonucleoproteins C1/C2	P07910	Nucleus	1	33.7

Heterogeneous nuclear ribonucleoprotein K	P61978	Nucleus	3	50.9
Histone H1.3	P16402	Nucleus	4	22.3
Histone H2A type 2-C	Q16777	Nucleus	2	14
Histone H1.5	P16401	Nucleus	2	22.6
Histone H2B type 1-K	O60814	Nucleus	7	13.9
Histone H3.3	P84243	Nucleus	4	15.3
Histone H4	P62805	Nucleus	6	11.4
Importin subunit beta-1	Q14974	Nucleus	2	97.1
Interleukin enhancer-binding factor 2	Q12905	Nucleus	1	43
Lysyl oxidase homolog 2	Q9Y4K0	Nucleus	1	86.7
m7GpppX diphosphatase	Q96C86	Nucleus	1	38.6
Mitotic checkpoint protein BUB3	O43684	Nucleus	3	37.1
Non-POU domain-containing octamer-binding protein	Q15233	Nucleus	2	54.2
Nuclear transport factor 2	P61970	Nucleus	2	14.5
Nucleolin	P19338	Nucleus	4	76.6
Nucleophosmin	P06748	Nucleus	6	32.6
Peptidyl-prolyl cis-trans isomerase E	Q9UNP9	Nucleus	1	33.4
Peptidyl-prolyl cis-trans isomerase H	O43447	Nucleus	1	19.2
Probable ATP-dependent RNA helicase DDX17	Q92841	Nucleus	2	80.2
Probable ATP-dependent RNA helicase DDX46	Q7L014	Nucleus	1	117.3
Probable global transcription activator SNF2L1	P28370	Nucleus	1	122.5
Programmed cell death protein 4	Q53EL6	Nucleus	1	51.7
Protein phosphatase 1 regulatory subunit 7	Q15435	Nucleus	1	41.5
Protein phosphatase methylesterase 1	Q9Y570	Nucleus	3	42.3
Protein RCC2	Q9P258	Nucleus	1	56
Pterin-4-alpha-carbinolamine dehydratase 2	Q9H0N5	Nucleus	1	14.4
Regulator of chromosome condensation	P18754	Nucleus	2	44.9
RuvB-like 1	Q9Y265	Nucleus	1	50.2
Serine/arginine-rich splicing factor 2	Q01130	Nucleus	4	25.5
Serine/threonine-protein phosphatase 5	P53041	Nucleus	1	56.8
Soluble lamin-associated protein of 75 kDa	Q9Y6X4	Nucleus	1	74.9
Spliceosome RNA helicase DDX39B	Q13838	Nucleus	4	49
Splicing factor, proline- and glutamine-rich	P23246	Nucleus	2	76.1
Splicing factor 3B subunit 3	Q15393	Nucleus	5	135.5
Squamous cell carcinoma antigen recognized by T-cells 3	Q15020	Nucleus	3	109.9
Transcription elongation factor SPT6	Q7KZ85	Nucleus	1	198.9
U5 small nuclear ribonucleoprotein 200 kDa helicase	O75643	Nucleus	1	244.4
X-ray repair cross-complementing protein 5	P13010	Nucleus	3	82.7
X-ray repair cross-complementing protein 6	P12956	Nucleus	4	69.8

60S ribosomal protein L6	Q02878	ER/Golgi	1	32.7
78 kDa glucose-regulated protein	P11021	ER/Golgi	2	72.3
A-kinase anchor protein 9	Q99996	ER/Golgi	1	453.4
AP-1 complex subunit gamma-1	O43747	ER/Golgi	2	91.3
AP-1 complex subunit mu-1	Q9BXS5	ER/Golgi	1	48.6
Coatomer subunit delta	P48444	ER/Golgi	1	57.2
Disintegrin and metalloproteinase domain-containing protein 10	O14672	ER/Golgi	2	84.1
Exostosin-1	Q16394	ER/Golgi	1	86.2
Hypoxia up-regulated protein 1	Q9Y4L1	ER/Golgi	3	111.3
Lipopolysaccharide-responsive and beige-like anchor protein	P50851	ER/Golgi	2	318.9
Pleckstrin homology domain	Q96JA3	ER/Golgi	1	58.2
Phospholipase D3	Q8IV08	ER/Golgi	2	54.7
Prostaglandin F2 receptor negative regulator	Q9P2B2	ER/Golgi	6	98.5
Protein disulfide-isomerase A3	P30101	ER/Golgi	4	56.7
Reticulon-1	Q16799	ER/Golgi	1	83.6
Trafficking protein particle complex subunit 4	Q9Y296	ER/Golgi	1	24.3
Ubiquitin carboxyl-terminal hydrolase isozyme L1	P09936	ER/Golgi	6	24.8
UDP-glucuronic acid decarboxylase 1	Q8NBZ7	ER/Golgi	1	47.5
Alpha-L-iduronidase	P35475	Lysosome	1	72.6
Peroxiredoxin-6	P30041	Lysosome	2	25
Ubiquitin carboxyl-terminal hydrolase 5	P45974	Lysosome	1	95.7
Arrestin-C	P36575	Unknown	1	42.8
Cysteine and histidine-rich domain	Q9UHD1	Unknown	1	37.5
UPF0553 protein C9orf64	Q5T6V5	Unknown	1	39
Uncharacterized protein KIAA0825	Q8IV33	Unknown	1	147.7
UPF0553 protein C9orf64	Q5T6V5	Unknown	1	39

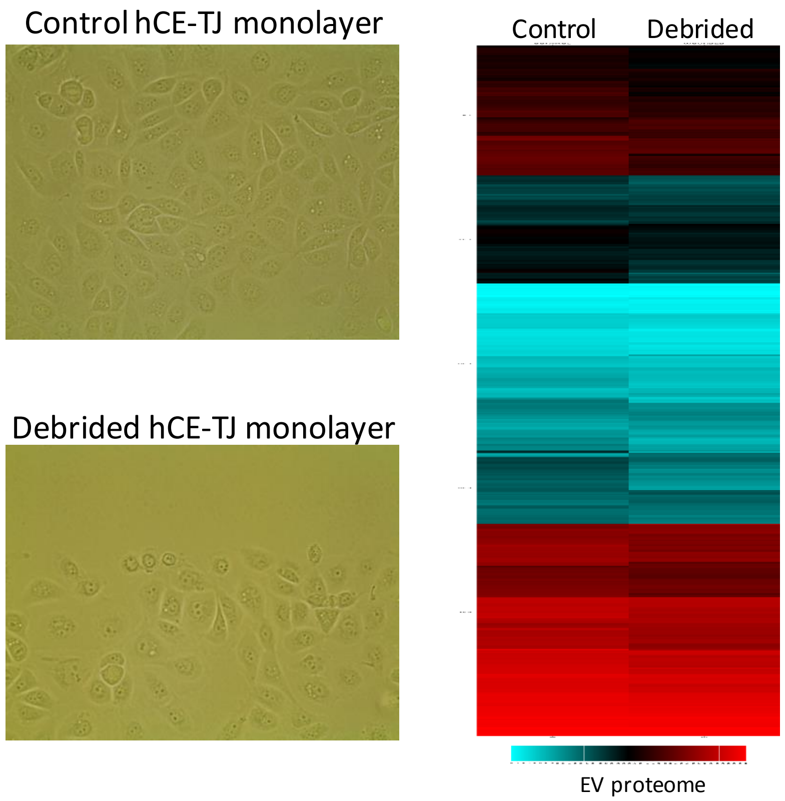

Figure S5. Effects of epithelial cell layer (hCE-TJ)-debridement on EV-protein cargo. Brightfield images of control and debrided hCE-TJ monolayers (*left*). Heat map showing the relative protein expression of hCE-TJ control EVs and debrided hCE-TJ-derived EVs (*right*).

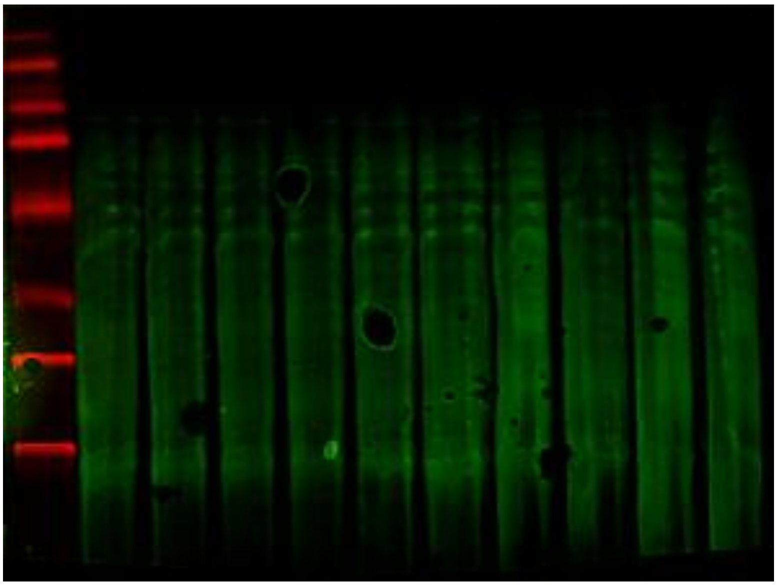

Figure S6. Full western blot of CD63 staining.

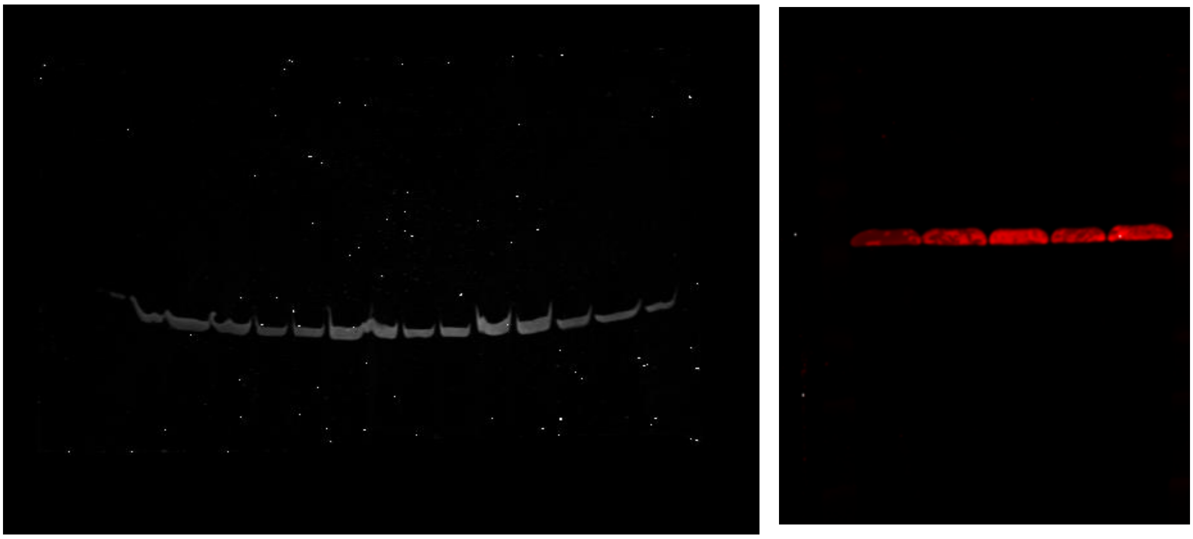

Figure S7. Full western blot of α-SMA staining.

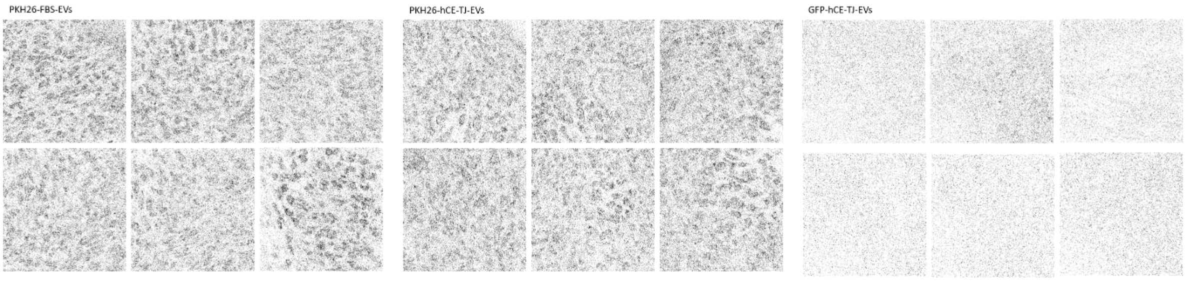

Figure S8. Black and white images of the particle analysis of the PKH26- or GFP-fluorescent signal for the EV-uptake study.

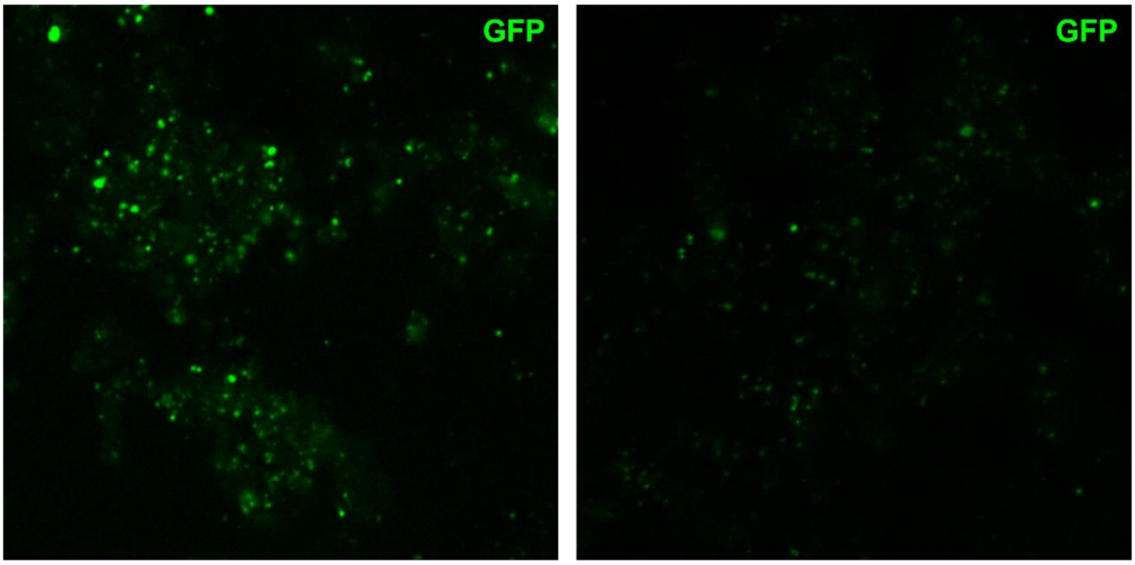

Figure S9. Stimulated emission depletion (STED) microscopy images of isolated GFP-expressing hCE-TJ-derived EVs.

© 2019 by the authors. Submitted for possible open access publication under the terms and conditions of the Creative Commons Attribution (CC BY) license (http://creativecommons.org/licenses/by/4.0/).
